# MmuPV1 E7’s interaction with PTPN14 delays Epithelial differentiation and contributes to virus-induced skin disease

**DOI:** 10.1371/journal.ppat.1011215

**Published:** 2023-04-10

**Authors:** James C. Romero-Masters, Miranda Grace, Denis Lee, Joshua Lei, Melanie DePamphilis, Darya Buehler, Rong Hu, Ella Ward-Shaw, Simon Blaine-Sauer, Nathalie Lavoie, Elizabeth A. White, Karl Munger, Paul F. Lambert

**Affiliations:** 1 McArdle Laboratory for Cancer Research, University of Wisconsin School of Medicine and Public Health, Madison, Wisconsin, United States of America; 2 Department of Developmental, Molecular and Chemical Biology, Tufts University School of Medicine, Boston, Massachusetts, United States of America; 3 Department of Pathology and Laboratory Medicine, School of Medicine and Public Health, University of Wisconsin, Madison, Wisconsin, United States of America; 4 Department of Molecular Biology and Microbiology, Tufts University School of Medicine, Boston, Massachusetts, United States of America; 5 Molecular Microbiology Program, Graduate School of Biomedical Sciences, Tufts University, Boston, Massachusetts, United States of America; 6 Department of Otorhinolaryngology, University of Pennsylvania, Philadelphia, Pennsylvania, United States of America; Penn State University School of Medicine, UNITED STATES

## Abstract

Human papillomaviruses (HPVs) contribute to approximately 5% of all human cancers. Species-specific barriers limit the ability to study HPV pathogenesis in animal models. Murine papillomavirus (MmuPV1) provides a powerful tool to study the roles of papillomavirus genes in pathogenesis arising from a natural infection. We previously identified Protein Tyrosine Phosphatase Non-Receptor Type 14 (PTPN14), a tumor suppressor targeted by HPV E7 proteins, as a putative cellular target of MmuPV1 E7. Here, we confirmed the MmuPV1 E7-PTPN14 interaction. Based on the published structure of the HPV18 E7/PTPN14 complex, we generated a MmuPV1 E7 mutant, E7^K81S^, that was defective for binding PTPN14. Wild-type (WT) and E7^K81S^ mutant viral genomes replicated as extrachromosomal circular DNAs to comparable levels in mouse keratinocytes. E7^K81S^ mutant virus (E7^K81S^ MmuPV1) was generated and used to infect FoxN/Nude mice. E7^K81S^ MmuPV1 caused neoplastic lesions at a frequency similar to that of WT MmuPV1, but the lesions arose later and were smaller than WT-induced lesions. The E7^K81S^ MmuPV1-induced lesions also had a trend towards a less severe grade of neoplastic disease. In the lesions, E7^K81S^ MmuPV1 supported the late (productive) stage of the viral life cycle and promoted E2F activity and cellular DNA synthesis in suprabasal epithelial cells to similar degrees as WT MmuPV1. There was a similar frequency of lateral spread of infections among mice infected with E7^K81S^ or WT MmuPV1. Compared to WT MmuPV1-induced lesions, E7^K81S^ MmuPV1-induced lesions had a significant expansion of cells expressing differentiation markers, Keratin 10 and Involucrin. We conclude that an intact PTPN14 binding site is necessary for MmuPV1 E7’s ability to contribute to papillomavirus-induced pathogenesis and this correlates with MmuPV1 E7 causing a delay in epithelial differentiation, which is a hallmark of papillomavirus-induced neoplasia.

## Author summary

Human papillomaviruses cause human cancers largely through virally encoded E6 and E7 oncogenes. A murine papillomavirus, MmuPV1, causes lesions in laboratory mice that mimic features of human papillomavirus-induced lesions including cancer. In this study, we investigated how the interaction of MmuPV1 E7 with the cellular tumor suppressor protein, PTPN14, contributes to viral pathogenesis using a MmuPV1 E7 mutant defective for interacting with PTPN14, E7^K81S^. We examined phenotypic differences between the E7^K81S^ and wild type MmuPV1 in tissue culture and in mice. The E7^K81S^ mutant genome was able to replicate as a nuclear plasmid in tissue culture. In vivo, E7^K81S^ mutant virus caused dysplastic lesions like wild type virus; however, they were smaller in size, arose later, and trended towards being less severe in their grade of neoplastic disease. The E7^K81S^-induced lesions showed evidence for increased E2F activation and reprogramming of suprabasal cells to support DNA synthesis similar to that seen in lesions induced by wild type virus. One clear molecular difference was that the E7^K81S^-induced lesions showed an expansion of cells expressing differentiation markers. Our study shows that an intact PTPN14 binding site is important for MmuPV1 E7’s ability to contribute to MmuPV1-induced skin disease.

## Introduction

Human papillomaviruses (HPVs) are major human pathogens that cause ~5% of the world’s total cancer burden including anogenital, head and neck, and skin cancers [1–4]. HPVs can be separated into cutaneous and mucosal groups based on which type of epithelia they primarily infect [5]. Cutaneous warts are generally benign lesions that are caused by infections with specific, cutaneous HPVs [6]. A subset of cutaneous HPVs, most prominently HPV5 and 8, cause cutaneous squamous cell carcinoma (cSCC) in individuals who are genetically predisposed to develop the rare skin disease, epidermodysplasia verruciformis (EV) [7–11]. HPV5 and 8 potentially also contribute to cSCC in other patients including immunocompromised individuals [12]. Mucosal HPVs infect the anogenital tract and oral mucosa [5]. Mucosal HPVs can be further categorized into ‘high-risk’ and ‘low-risk’ types [5]. The low-risk mucosal HPVs are associated with benign genital warts [5,13]. In contrast, high-risk mucosal HPVs cause lesions that can progress to anogenital and head and neck cancers [5,13].

The ability of mucosal high-risk HPVs to promote carcinogenesis is driven by the virally encoded E5, E6, and E7 oncogenes [14,15]. The oncogenic activities of the high-risk mucosal HPV E6 and E7 proteins have been extensively studied in tissue culture and transgenic animal-based models [14,16–20]. The best studied oncogenic activities of E6 and E7 are E7’s inactivation of the retinoblastoma tumor suppressor (pRB), which leads to elevated E2F activity and unscheduled cellular DNA synthesis, and E6’s inactivation the p53 tumor suppressor [21–23]. A large number of additional potential cellular target proteins of E6 and E7 have been identified [24,25]. Many cutaneous HPV E6 and E7 proteins also have oncogenic activities in tissue culture and transgenic animal-based systems but they have been less well studied mechanistically [26–37]. Of note, some cutaneous HPV E6 proteins target the NOTCH and TGF-β tumor suppressive signaling pathways instead of p53 [38–41].

Because of the species specificity of papillomaviruses, it has not been possible to investigate the contributions of HPV E6 and E7 to viral pathogenesis *in vivo* in the context of a natural infection. The murine papillomavirus (MmuPV1), discovered in 2011, naturally infects laboratory mice [42]. The MmuPV1 model system now provides an opportunity to study the activities of the viral proteins in the context of natural infection in a genetically tractable animal species [43,44]. MmuPV1 can infect both cutaneous and mucosal tissues and causes neoplastic and malignant lesions that recapitulate many features of HPV-associated human disease [43,45–49]. MmuPV1 encodes E6 and E7 proteins but, similar to cutaneous HPVs, lacks an E5 gene [50,51]. The MmuPV1 E6 and E7 genes are each required for viral pathogenesis [38,52]. Like many cutaneous HPV E6 proteins, the MmuPV1 E6 protein does not directly target p53. Rather, it subverts the TGF-β and NOTCH signaling pathways [38]. Like HPV E7 proteins, MmuPV1 E7 binds to pRB; however, the means by which it does so differs from most HPV E7 proteins [52]. Instead of binding the B box of pRB’s pocket domain through an LXCXE motif found in the N terminus of most HPV E7 proteins, MmuPV1 E7 binds a domain in the C-terminus of pRB through amino acids located in its own C-terminus [52]. This alternative mode of binding pRB is shared with a subset of HPV E7 proteins that also lack LXCXE domains [53,54]. MmuPV1 E7’s mode of binding to pRB does not result in increased E2F transcription factor activity [52]. Mutational studies indicate that MmuPV1 E7’s interaction with pRB promotes viral pathogenesis, but it does not fully account for the contributions of MmuPV1 E7 to viral disease [52]. This has led us to investigate other activities of the MmuPV1 E7 protein.

In a previous study, we identified the cellular non-receptor tyrosine phosphatase PTPN14 as a putative cellular target of MmuPV1 E7 [52]. PTPN14 is a well-known target of many HPV E7 proteins and is considered a tumor suppressor in part because loss-of-function mutations in PTPN14 are associated with increased cancer risk [55–58]. A tumor suppressive activity of PTPN14 is to inhibit YAP activity [59]. High-risk mucosal HPV E7s interact with and cause PTPN14 degradation [55,56,60,61]. HPV E7 mutant proteins that are unable to bind and degrade PTPN14 retain the ability to bind and inhibit pRB, demonstrating that these are separable activities [60,61]. Previous work has shown that high-risk HPV E7 proteins’ interactions with PTPN14 are important in maintaining basal cell identity in 3 dimensional cultures of human keratinocytes and contribute to the ability of E7 to inhibit epithelial differentiation in monolayer cultures of keratinocytes [60–62]. The importance of this interaction in viral pathogenesis *in vivo* has not been evaluated to date. The MmuPV1 mouse model provides us an opportunity to examine the role of this interaction in papillomaviral pathogenesis.

In this study, we confirmed that MmuPV1 E7 interacts with PTPN14 and showed a trend towards causing reduced steady levels of PTPN14. We generated a MmuPV1 E7 mutant, E7^K81S^, that is defective in its interaction with PTPN14 and characterized its phenotype *in vivo* using the MmuPV1 skin infection mouse model [49]. We found that, like WT MmuPV1, E7^K81S^ MmuPV1 was competent in infecting mouse keratinocytes and in establishing and maintaining its DNA genome as an extrachromosomal, circular, double-stranded, DNA which are features of the early stage of the viral life cycle. In mice infected with E7^K81S^ MmuPV1, skin lesions arose at a similar frequency as in mice infected with WT-MmuPV1, but the lesions arose significantly later and were significantly smaller in size at the 6-month end-point. The lesions also trended towards being less severe in their grade of neoplastic disease. E7^K81S^ MmuPV1-induced lesions showed evidence for the late stage of the viral life cycle based upon expression of the major capsid protein, L1, and amplification of the viral genome. Lateral spread of infectious disease in mice infected with E7^K81S^ MmuPV1 provided evidence that this mutant virus retains the ability to generate infectious progeny. In the E7^K81S^ MmuPV1-induced lesions, we observed increased expression of the *Mcm7* protein, which is upregulated by E2F, and reprogramming of suprabasal cells to undergo DNA synthesis (based upon BrdU-incorporation), much like we have previously documented for WT MmuPV1 [52]. One striking difference between WT and E7^K81S^ MmuPV1-induced lesions was that, in the latter, we observed an expansion of epithelial cells expressing markers of keratinocyte differentiation (K10 and Involucrin). Based on these results we conclude that MmuPV1 E7’s binding to PTPN14 contributes to viral pathogenesis and to a delay in keratinocyte differentiation, which is a common feature of papillomavirus-induced disease.

## Results

### MmuPV1 E7 interacts with cellular Protein Tyrosine Phosphatase Non-Receptor Type 14 (PTPN14)

**Fig 1:**
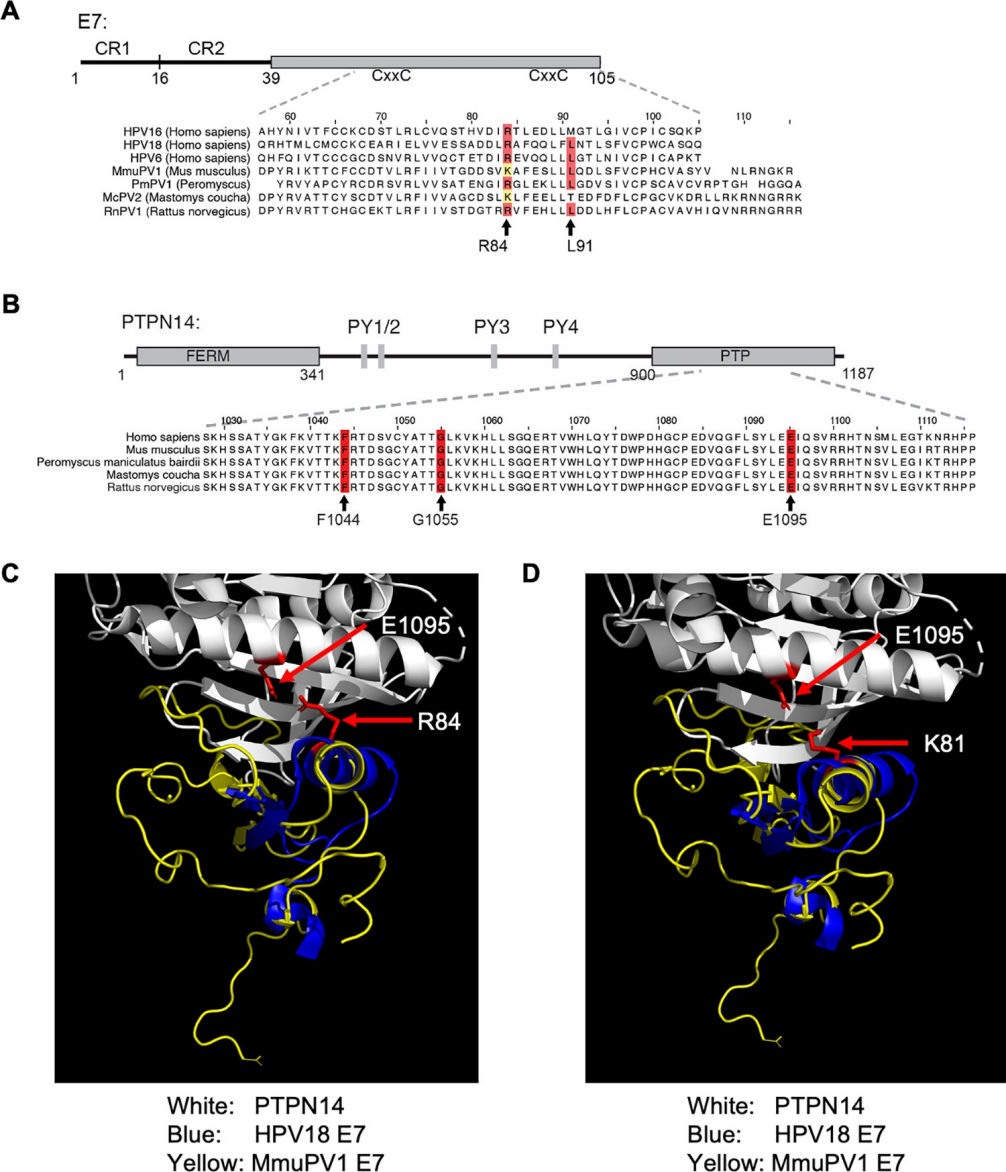
*In Silico* analysis of amino acid sequence and protein structure identifies key amino acids for MmuPV1 E7’s interaction with PTPN14. (A) Schematic of a representative HPV E7 protein. Papillomavirus E7 proteins contain conserved regions 1 and 2 (CR1, CR2) and a highly structured C-terminus organized around two zinc binding motifs (CxxC). Enlargement indicates an amino acid sequence alignment of the C-termini of several human and rodent papillomavirus E7 proteins. R84 and L91 are two amino acids in HPV18 E7 that make contact with PTPN14 as determined by the HPV18 E7-PTPN14 crystal structure [63]. Identity with these amino acids is indicated in red. An arginine corresponding to HPV18 R84 is conserved among many papillomavirus E7 but is replaced by lysine in at least two rodent papillomavirus E7 (yellow highlight). (B) Schematic of Homo sapiens (Hs)PTPN14 including its 4.1, ezrin, radixin, moesin (FERM) domain, protein tyrosine phosphatase (PTP) domain, and PxxY (PY) motifs identified in [87,88]. Enlargement indicates an amino acid sequence alignment of a segment of the PTP domains of human and selected rodent PTPN14 proteins. F1044, G1055, and E1095 are three amino acids in PTPN14 that make contact with HPV18 E7 as determined by the HPV18 E7-PTPN14 crystal structure [63], and these are highlighted in red. (C & D) Threading analysis using the structure information from the interaction between PTPN14 (white) and HPV18 E7 (blue) (C) was used to predict the structure of the PTPN14 (white) and MmuPV1 E7 (yellow) (D). Critical amino acids for the interaction between E7 and PTPN14 are highlighted in red with E1095 of PTPN14 indicated in both panels (C & D), and arginine (R) 84 in HPV18 E7 (C) and lysine (K) 81 in MmuPV1 (D). Red arrows point to the amino acids highlighted in the images. Only the regions within PTPN14 and E7 that interact are shown.

Our previously published affinity purification mass spectrometry analysis of the MmuPV1 E7 protein interactome identified PTPN14 as a host protein that interacts with MmuPV1 E7 [52]. Structural studies using HPV18 E7 identified specific, amino acid (AA) residues in the C-terminal half of E7 (e.g., arginine (R) 84, glutamine (Q) 87, and leucine (L) 91) in HPV18 E7 (Fig 1A) that make electrostatic and hydrophobic interactions with well-conserved amino acids in PTPN14 (Figs 1B and S1B) [63]. The arginine corresponding to R84 of HPV18 E7 is well-conserved across cutaneous and mucosal HPVs (Figs 1A and S1A). Mutation of that arginine to a serine (S) in several different HPV E7 proteins reduced the ability of each HPV E7 to co-immunoprecipitate with PTPN14 [61].

We compared the AA sequence of HPV18 E7 to that of MmuPV1 E7 (Fig 1A). In MmuPV1 E7, the lysine (K) at position 81 (K81) corresponds to HPV18 E7 R84. Arginine and lysine residues both have positively charged side chains. While most papillomavirus E7 proteins have arginine residues at this position, a lysine is also present at the same aligned position of the E7 protein encoded by McPV2, which infects another rodent species, *Mastomys coucha* (Southern multimammate mouse) (Fig 1A). We also aligned the region of PTPN14 that binds to HPV E7 and found that the PTPN14 AA sequence in this region was highly conserved across species (Fig 1B). Threading of MmuPV1 E7 primary AA sequence to the known tertiary structure of HPV18 E7 (PDB: 6IWD) by the Iterative Threading ASSEmbly Refinement (I-TASSER) server suggests that the C-terminal domains of MmuPV1 E7 and HPV18 E7 have similar tertiary structures with a shared alpha helix interface with human PTPN14 (Fig 1C and 1D). We further examined if there are potential differences in how K81 in MmuPV1 E7 interacts with the glutamic acid at AA 1095 (E1095) in PTPN14 compared to R84 in HPV18 E7 (Fig 1C). The orientation of K81 in MmuPV1 E7 and R84 in HPV18 E7 with E1095 in PTPN14 was similar (Fig 1C and 1D). However, the predicted distance between the K81 in MmuPV1 E7 and E1095 in PTPN14 was predicted to be larger (~4.4 Å) than the distance between R81 in HPV18 E7 and E1095 in PTPN14 (~3 Å), which may impact binding (Fig 1C and 1D).

**Fig 2:**
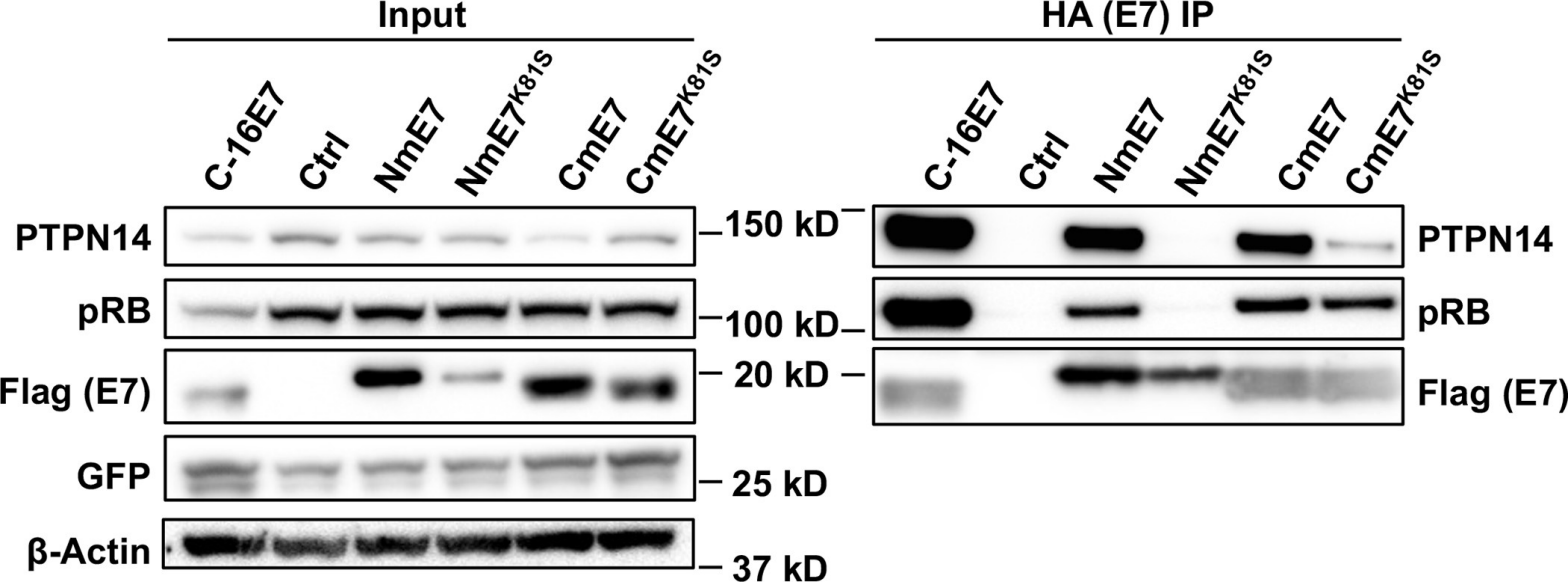
A lysine at amino acid position 81 in MmuPV1 E7 is critical for the interaction with PTPN14. NIH 3T3 cells were transfected with either empty vector (N) or plasmid constructs that express N-terminally and C-terminally HA/FLAG epitope-tagged HPV16 E7, WT MmuPV1 E7, or MmuPV1 E7^K81S^ mutant. MmuPV1 E7 constructs were either N- or C-terminally tagged as indicated. Cells were harvested and subjected to immunoprecipitation with HA antibodies. For HA IP, 1 mg of HPV16 E7 and 3 mg of other lysates was used and 100 ug WCL on 4–12% mini gels was used for input Western blot analysis. Whole cell lysates (Input) and HA IP eluates were subjected to western blot analysis using antibodies against PTPN14, pRb, FLAG (E7), Actin, or GFP (transfection control). Left panel on blot is the same images labeled Experiment 1 in S2 Fig.

To validate the interaction of MmuPV1 E7 with PTPN14, we transfected mouse NIH 3T3 fibroblasts with plasmids expressing C-terminally HA/FLAG epitope-tagged HPV16 E7 (as a positive control) or either N-terminally or C-terminally HA/FLAG epitope-tagged MmuPV1 E7 and performed immunoprecipitations using anti-HA antibody on transfected cell protein lysates. HPV16 E7, like HPV18 E7, interacts with and degrades PTPN14 and served as our positive control for binding and degradation [55]. The immunoprecipitated proteins were then analyzed by western blot using antibodies to either FLAG tag (to detect the E7 proteins) or PTPN14 (Fig 2). N-terminally and C-terminally epitope tagged MmuPV1 E7 were able to bind to PTPN14. To determine whether MmuPV1 E7 K81 is important for binding to PTPN14, we generated a MmuPV1 E7 mutant in which K81 was replaced with a serine (S) residue (K81S). This matches the R84S mutation in HPV18 E7 that disrupted its interaction with PTPN14 [61]. As described above, PTPN14 co-immunoprecipitated with wild type MmuPV1 E7 in NIH3T3 cells, but we observed reduced co-immunoprecipitation of PTPN14 with C-terminally tagged MmuPV1 E7^K81S^ (Fig 2), similar to what has been reported for the HPV18 E7^R84S^ mutant protein [61]. The steady state levels of the C-terminally tagged MmuPV1 E7^K81S^ mutant were similar to wild type C-terminally tagged MmuPV1 E7. Co-immunoprecipitation of PTPN14 with N-terminally tagged MmuPV1 E7^K81S^ was also decreased compared to its interaction with WT MmuPV1 E7. However, steady-state levels of N-terminally tagged E7^K81S^ were also lower than WT MmuPV1 E7 levels. Moreover, the K81S mutation did not appear to impair the general integrity of the C-terminal MmuPV1 E7 domain since the C-terminally tagged MmuPV1 E7^K81S^ mutant retained its ability to bind pRB, which we previously mapped to an adjacent C-terminal AA. The N-terminally tagged E7^K81S^ mutant showed reduced binding to pRB, perhaps as a result of the lower levels of input protein. In several replicate experiments using E7 tagged at the C-terminus, there was a trend towards lower steady-state levels of PTPN14 in NIH3T3 cells that were transiently transfected with WT MmuPV1 E7, but not in MmuPV1 E7^K81S^-transfected cells (S2 Fig). In summary, these results show that MmuPV1 E7 can bind to the cellular protein PTPN14 and that the K81S mutation greatly reduces that binding.

### The MmuPV1 E7^K81S^ mutant does not impair viral genome replication/maintenance

**Fig 3:**
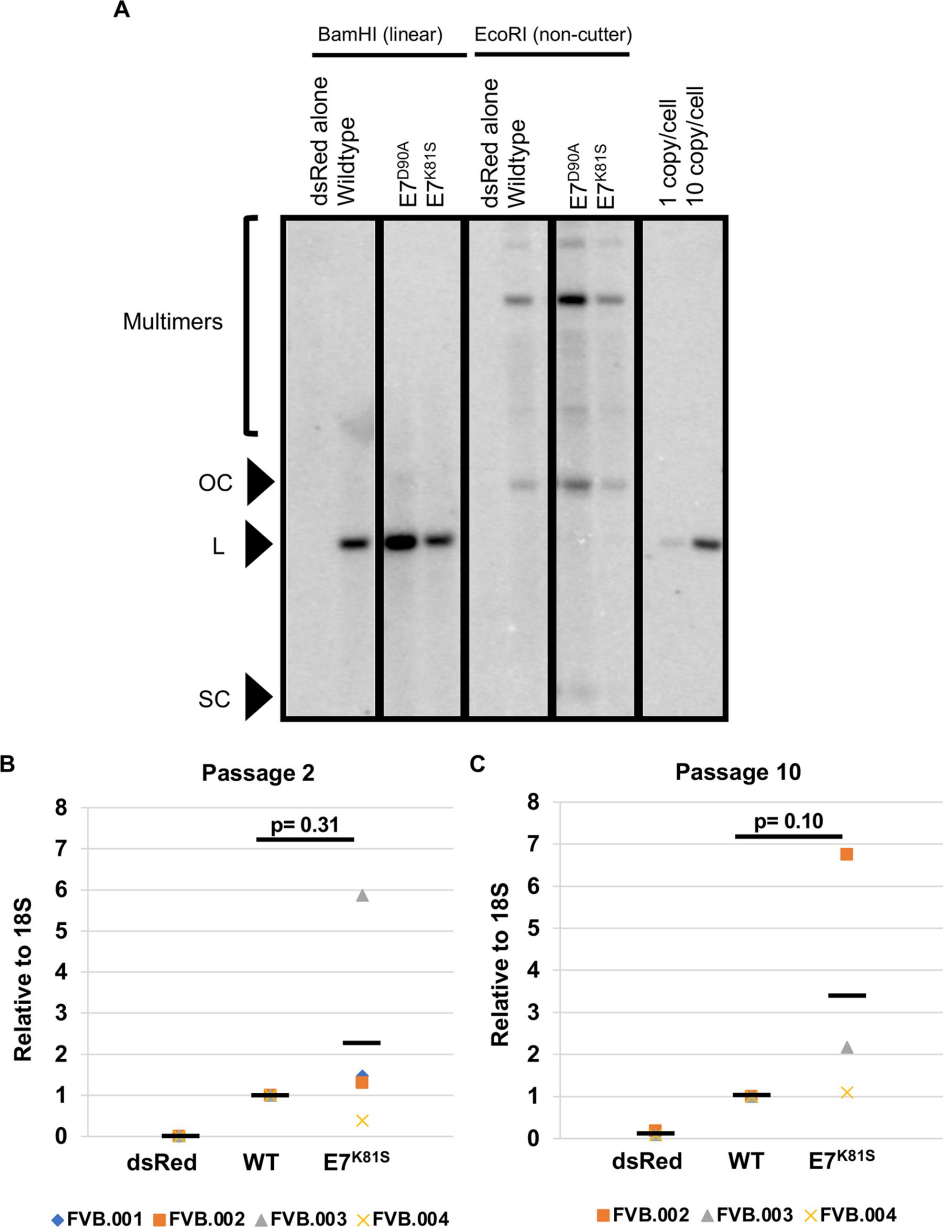
MmuPV1 E7^K81S^ mutant does not impair viral genome maintenance in basal keratinocytes. (A) Early passage mouse keratinocytes (MKs) isolated from neonates were transfected with dsRed alone or in combination with WT, E7^D90A^, or E7^K81S^ re-circularized viral genomes. DNA was isolated and restriction digested with a linearizing enzyme (BamHI) or non-cutter (EcoRI). Southern blot analysis was performed using an MmuPV1 genome probe included a standard of 1 copy/cell and 10 copy/cell to estimate copy number level per cell. The positions of open circular (OC), linear (L), and supercoiled (SC) genomes are indicated. The different panels are derived from the same gel and have been sampled at the same exposure. An uncropped version of Fig 3A is provided in S3A Fig. (B and C) DNA isolated from transfected MKs were subjected to qPCR analysis using primers for the viral E2 gene and mouse 18S gene. Viral genome copy number was normalized to total DNA content as determined by levels of the 18S gene and relative copy number is indicated after normalization to the level of genomes in WT MmuPV1 transfected cells. Three different transfected MK cell populations are shown. Wilcoxon rank sum test was performed on the different conditions to assess statistical significance. Black bars indicate means for each condition.

Having established that amino acid K81 in MmuPV1 E7 is required for binding to PTPN14, we sought to characterize the phenotype of the MmuPV1 E7^K81S^ mutant *in vivo*. First, we introduced the E7^K81S^ mutation into a plasmid containing the full-length, MmuPV1 DNA genome and assessed the ability of WT and mutant genomes to replicate as extrachromosomal DNA elements in primary murine keratinocytes, the natural host cell for MmuPV1. Early passage mouse keratinocytes were transfected with re-circularized, wild type, E7^K81S^ mutant, or E7^D90A^ mutant (defective for pRB binding) MmuPV1 genomes that had been released from the bacterial plasmid vector and recircularized. Following drug selection for a co-transfected plasmid conferring blasticidin resistance, DNA from transfected cells was subjected to Southern blot analysis using MmuPV1-specific DNA probes (Figs 3A and S3A). The wild type, E7^K81S^, and E7^D90A^ mutant MmuPV1 genomes were maintained as extrachromosomal circular DNAs in the mouse keratinocytes with average copy numbers of ~10 copies/cell. We further performed qPCR to assess differences in copy number between the wild type (WT) and E7^K81S^ mutant viral genomes in at least three different populations of transfected mouse keratinocytes at both early (Passage 2) and later (Passage 10) passages after transfection (Fig 3B and 3C). We detected a slight (~2 fold at passage 2 and ~3 fold at passage 10) increase in E7^K81S^ mutant viral genomes compared to wild type viral DNA, but this difference did not reach statistical significance. We observed no alteration in the growth rates of the cells transfected with the recircularized wild type vs E7^K81S^ MmuPV1 genomes. These results show that the E7^K81S^ mutant expressing viral genome is replication competent. This indicated to us that *in vivo* pathogenesis studies using this mutant were feasible.

### MmuPV1 E7’s interaction with PTPN14 contributes to viral pathogenesis

**Fig 4:**
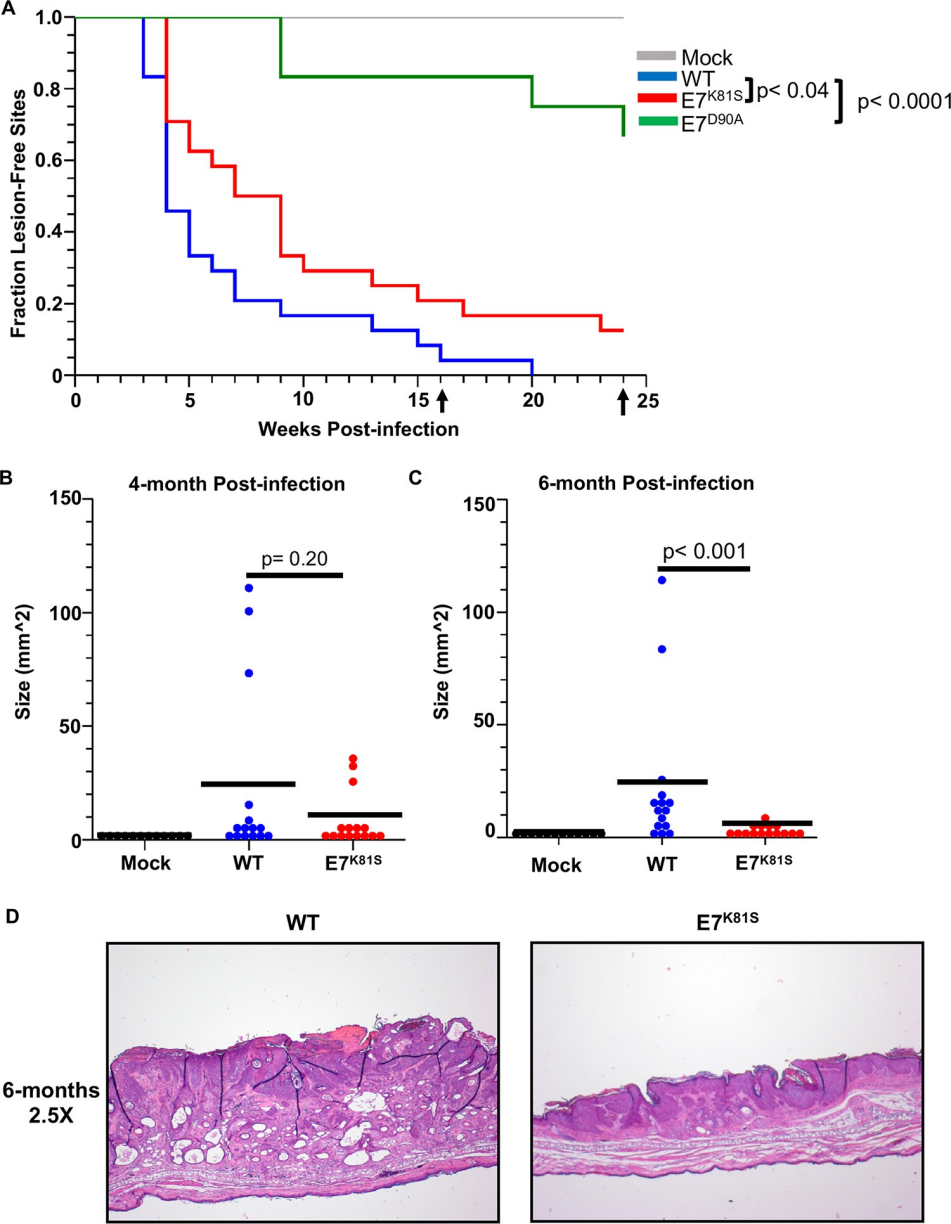
E7^K81S^ MmuPV1 induces lesions at a slower rate and the lesions are smaller in size than those induced by WT MmuPV1. **(A)** Mice were monitored for papilloma development over a 6-month period every two weeks following infection. Each ear was considered a site of infection and the week post-infection that a lesion developed was recorded. A Kaplan-Meier curve was generated using the week post-infection versus fraction of lesion-free sites across all animals. Data was subjected to Log-Rank test to assess statistical significance (p<0.05 was considered significant). **(B&C)** Lesions were measured at the time of collection for area of lesion size (depth was excluded from measurements). Data was plotted as dot plots with each site measured represented by a single dot. Wilcoxon-rank sum analysis was performed on the data to assess statistical significance (p<0.05 was considered significant). Black bars indicate the mean for each condition. **(D)** Images of H&E-stained tissue sections at 2.5X magnification to show size differences of lesions at 6-months post-infection.

To test whether MmuPV1 E7^K81S^ virus could promote the development of lesions *in vivo* similar to wild type MmuPV1, we monitored development of skin papillomas in mice infected with wild type or E7^K81S^ mutant MmuPV1. We generated stocks of viruses in 293FT cells transfected with recircularized recombinant MmuPV1 genomic DNA (wild-type or mutant), which had been released from the bacterial vector, along with a plasmid that expresses codon optimized MmuPV1 L1 and L2 capsid genes, as per our established protocol [64]. Virus made by this method is commonly referred to as quasivirus to distinguish it from virus made in natural host cells. For simplicity, we refer to this virus as MmuPV1. In addition to making wild type and E7^K81S^ MmuPV1, we also made E7^D90A^ MmuPV1, which encodes a mutant E7 protein defective for binding to pRB [52], in order to compare the efficiency of E7^K81S^ MmuPV1 to cause disease to another mutant MmuPV1 defective in a different interaction. We titered the virus by Southern blot analysis to determine viral genome equivalents of each stock (VGE). To verify that the viruses generated were infectious, we monitored expression of viral E1^E4 transcripts in mouse JB6 keratinocytes 48 hours post exposure to quasivirus (S3B Fig). Ears of FoxN/nude mice were scarified and then exposed to 10^8^ VGE of mutant or wild type MmuPV1 and monitored for development of lesions over a six-month period. By this endpoint, 100% (24/24) of sites exposed to wild type (WT) MmuPV1 developed lesions, whereas 87% (21/24) of sites exposed to E7^K81S^ MmuPV1 developed lesions (Table 1). This difference was not significantly different (p = 0.23). In contrast, consistent with our prior studies [52], E7^D90A^ MmuPV1 infected mice developed significantly fewer lesions (33.3%, 4/12 sites) than wild type MmuPV1 infected mice (p<0.0001) (Table 1). To ensure that lesions were induced by the E7^K81S^ virus, DNA isolated from formalin-fixed paraffin-embedded tissue was used for sequencing of the MmuPV1 E7 gene. We found that DNA from the E7^K81S^ infected lesions contained the correct mutation (S4 Fig). We also monitored time to onset of disease and size of lesions arising in these mice (Fig 4). Here we saw significant differences in pathogenesis comparing E7^K81S^ MmuPv1 to wild type MmuPV1. Warts developed more slowly in the E7^K81S^ infected animals compared to wild type infected animals (p < 0.04). Consistent with our previous findings a greater delay was observed in E7^D90A^ MmuPV1 infected mice (Fig 4A) [52]. The lesions (scored as area) caused by E7^K81S^ MmuPV1 were smaller than those caused by wild type MmuPV1 four months post-infection and this difference was statistically significant at the six-month endpoint (p < 0.001) (Fig 4B and 4C). Although there was ~3-fold decrease in average lesion size between 4-months and 6-months post-infection in the E7^K81S^-induced lesions, this difference did not reach statistical significance. The difference in the size of wild type vs mutant lesions could be observed on H&E-stained, formalin-fixed paraffin embedded tissue slides with representative low magnification images (2.5X) (Fig 4D). Representative images of mouse ears from mock, wild type, or E7^K81S^ infected, with lesions circled, show gross differences in lesion size between wild type and E7^K81S^ infected animals (S5 Fig).

**Table 1:** Wart Incidence of infected Nude mice.

Virus	Number of Sites Infected	Number of sites with Lesions	Percent of sites with Lesions
**Mock**	24	0	0
**WT**	24	24	100
**E7** ^**K81S**^	24	21	87.50^ns^
**E7** ^**D90A**^	12	4	33.33*

Fisher’s exact statistical test was performed. ^ns^ E7^K81S^ vs WT, p = 0.23. * E7^D90A^ vs WT, p< 0.001.

**Fig 5:**
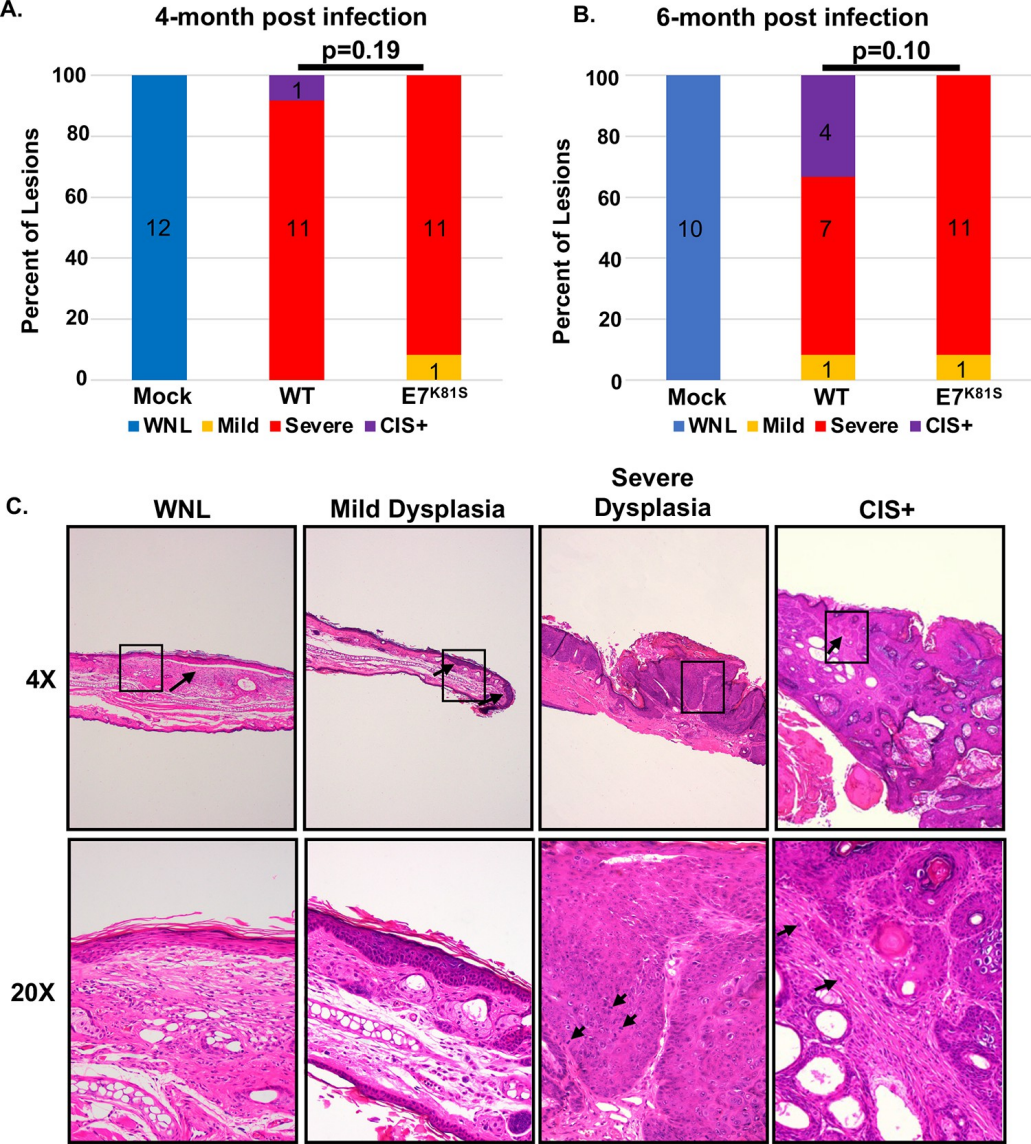
E7^K81S^ MmuPV1 causes severe dysplasia comparable to WT MmuPV1. **(A&B)** H&E tissue sections were scored for disease severity by two pathologists in a blinded fashion. Mock (uninfected) treated mice showed skin within normal limits (WNL). WT and E7^K81S^-infected mice all showed a range of mild dysplasia, severe dysplasia, and carcinoma in situ (CIS+) as indicated. Data was subjected to Wilcoxon-rank sum analysis to assess statistical significance (p<0.05 was considered significant). **(C)** Representative images of different disease severity are shown at low (4X) and high (20X) magnification. **WNL:** Normal ear skin with the arrow indicating a prior scarification site. At high magnification, no epithelial multilayering or cytologic atypia is observed. **Mild Dysplasia:** Mild thickening of surface epithelium (arrows). At high power, proliferation at the base of the epithelium with mild nuclear crowding, hyperchromasia and koliocytes is observed. There is preservation of epithelial maturation in the top half of the epithelium. **Severe Dysplasia:** Marked thickening and papillomatosis of surface epithelium. At high power, nuclear crowding and marked nuclear atypia and numerous mitotic figures (arrows) are present; surface maturation is minimal or absent. **CIS+**: In the background of severe dysplasia, an irregular area with bright eosinophilic keratinization is present. At high magnification, single epithelial cells in the stroma are present. These features are highly suspicious for nascent microinvasive squamous cell carcinoma.

To test whether MmuPV1 E7^K81S^ causes less severe disease than wild type MmuPV1, mouse ears were collected at the four-month time point and the six-month endpoint post-infection, fixed in 4% paraformaldehyde, paraffin-embedded, sectioned into 5 μm sections, and stained with hematoxylin and eosin (H&E). Histological sections were analyzed by two pathologists (DB and RH) blinded to sample identity and classified as mild or severe dysplasia, carcinoma in situ (CIS), or invasive squamous cell carcinoma (cSCC). While mice infected with wild type MmuPV1 developed CIS, which represents early invasive disease, no mice infected with E7^K81S^ MmuPV1 progressed to this advanced stage of neoplasia by 6 months post-infection. The difference in distribution of disease states in the two populations did not reach statistical significance (p = 0.1) (Fig 5). Our data show that MmuPV1 can promote skin lesion formation in the absence of an interaction between E7 and PTPN14, but the lesions develop more rapidly and become larger when E7 can bind to PTPN14.

### Readouts for the productive phase of the papillomaviral life cycle are not altered in lesions arising from infection with E7^K81S^ mutant quasivirus

**Fig 6:**
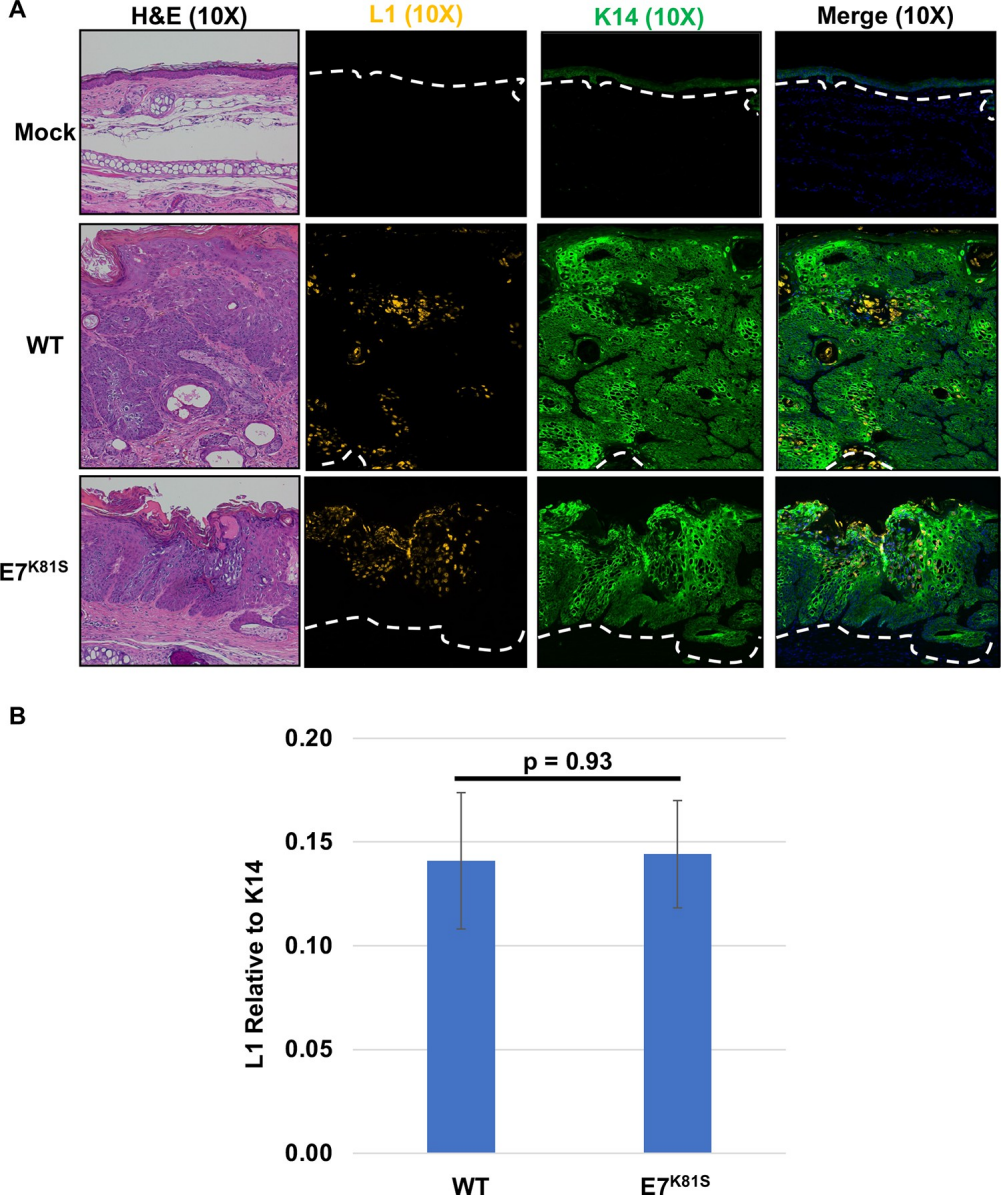
MmuPV1 E7^K81S^ mutant does not impair capsid protein production. **(A)** FFPE tissue sections were subjected to immunofluorescence (IF) analysis using a tyramide signal amplification (TSA) system using antibodies against K14 (green) and the viral capsid protein L1 (yellow). Representative images are shown for K14, L1, and merged (which includes Hoechst stain) at 10X magnification. A 10X image of the H&E is also shown. Dashed white lines indicate basement membrane of epithelial tissues. **(B)** IF images were quantified using ImageJ to determine area of lesion that stained positive for L1 and K14 using 9 images per condition. L1 signal was normalized to K14 signal. Standard error is shown. Wilcoxon Rank Sum test was performed to assess statistical significance and p-value is shown.

We monitored several biomarkers of the viral life cycle in the lesions arising in mice infected with wild type or E7^K81S^ MmuPV1: MmuPV1 L1 capsid protein expression, MmuPV1 genome amplification, and MmuPV1 gene transcription. To detect L1 protein, we performed immunofluorescence using Tyramide Signal amplification system (TSA) on Formalin-fixed paraffin embedded (FFPE) tissues for the MmuPV1 L1 protein and K14, a marker of basal keratinocytes. L1 capsid protein was produced at comparable levels in the E7^K81S^ MmuPV1-induced lesions and in lesions arising in wild type MmuPV1-infected animals (Figs 6 and S6). Consistent with a prior study [65], L1 protein was produced both in the poorly differentiated (K14-positive–see Fig 6) and in more well-differentiated (K14-negative, see S6 Fig) compartments of stratified squamous epithelium in wild type MmuPV1-induced lesions. This was also true for E7^K81S^ MmuPV1-induced lesions. Quantitative analysis of IF images for L1 and K14 also showed no significant difference in the amount of L1 signal relative to K14 between WT- and E7^K81S^-induced lesions (Fig 6B). These results indicate that the expression of the major viral capsid protein, L1, which is necessary for production of progeny virions, is not impaired in the E7^K81S^ MmuPV1-induced lesions.

**Fig 7:**
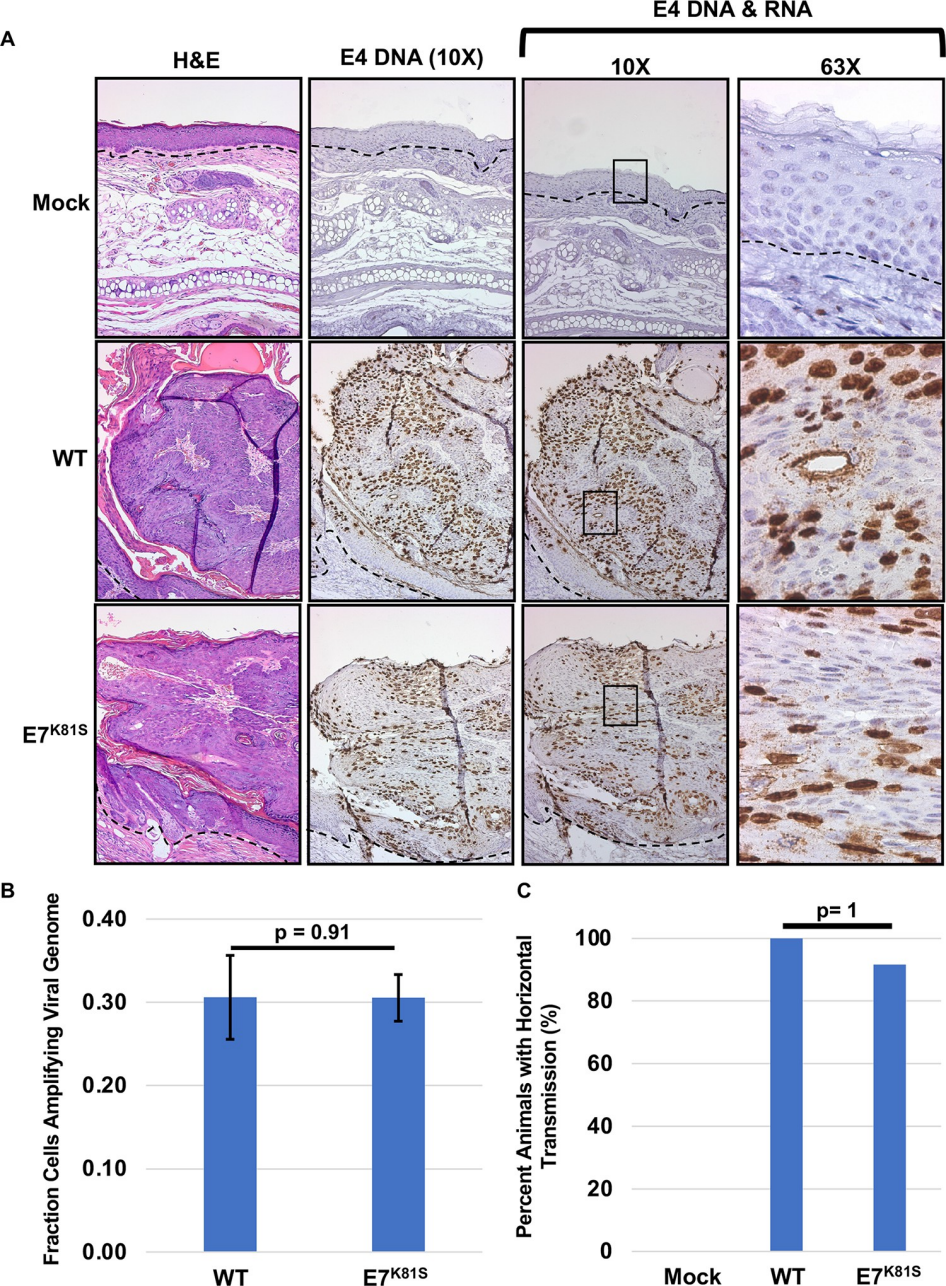
MmuPV1 E7^K81S^ mutant quasivirus produces viral E4 transcripts and genome amplification in lesions to the same degree as WT quasivirus. **(A)** FFPE tissue were subjected to in situ hybridization with a probe that detects both E4 RNA and DNA using the RNAscope technology. 10X images are shown for H&E. Representative high (63X) and low (10X) magnification images are shown for RNA/DNAscope and DNAscope (RNAse treated section) to detect viral E4 transcripts and viral genome amplification. Dashed black lines indicate basement membrane of epithelial tissues. **(B)** The fraction of cells going through viral genome amplification within a lesion is shown by determining the number of nuclei and number of positive staining nuclei using an ImageJ plugin developed by the Lab of Dr. David Ornelles (Wake Forest) for 18 fields of view. Ratio of positive staining cells to the total number of nuclei was determined and average shown. Standard error is shown. Wilcoxon Rank Sum test was performed to assess statistical significance and p-value is shown. **(C)** Upon euthanasia, animals were examined for development of warts/lesions at secondary sites of infection (sites other than ears). The percentage of animals that developed warts/lesions was calculated and shown. Fischer’s Exact Test was used to assess statistical significance.

To assess viral DNA amplification and viral transcription in wild type and E7^K81S^ MmuPV1-infected tissue, we performed *in situ* hybridization using RNAscope. We previously established that RNAscope probes can detect both MmuPV1 DNA and RNA [45,51]. RNAscope therefore serves as a sensitive method for detecting both viral DNA genomes present in the nucleus (presumably in cells supporting the late-stage amplification of the viral DNA) and viral RNA transcripts present in the cytoplasm. FFPE tissue slides were either untreated or treated with RNAse (for viral DNA only detection) prior to detecting the viral nucleic acids with MmuPV1-specific probes to the E4 region of the viral genome. E4 sequences are present in many abundant viral transcripts arising from both early and late viral promoters [51]. E7^K81S^ MmuPV1-induced lesions expressed viral transcripts (cytoplasmic staining) containing the E4 gene comparable to that observed in wild type MmuPV1-induced lesions (Fig 7A, see insets). Likewise, the nuclear signal for amplified viral DNA was comparable between the mutant and wild type infected lesions and the fraction of cells going through genome amplification was similar (Fig 7A and 7B). Collectively, these findings indicate that MmuPV1 DNA is amplified and MmuPV1 transcripts that contain E4 are expressed at comparable levels in wild type and E7^K81S^ MmuPV1-induced lesions (Fig 7A).

Nude mice transmit MmuPV1 horizontally by grooming, causing lesions to develop at secondary cutaneous sites. To assess whether lesions were producing infectious virus, mice were examined upon euthanasia for the development of warts/lesions at other cutaneous sites (skin tissue other than ears). All animals in wild type (12/12 animals, 100%) and nearly all animals in E7^K81S^ (11/12 animals, 91.67%) developed warts/lesions at other cutaneous sites (Fig 7C). This demonstrates that infectious virus is being produced in both the wild type and E7^K81S^ infected lesions.

### Both WT and E7^K81S^ MmuPV1-induced lesions show evidence for elevated E2F activity and increased suprabasal DNA synthesis

**Fig 8:**
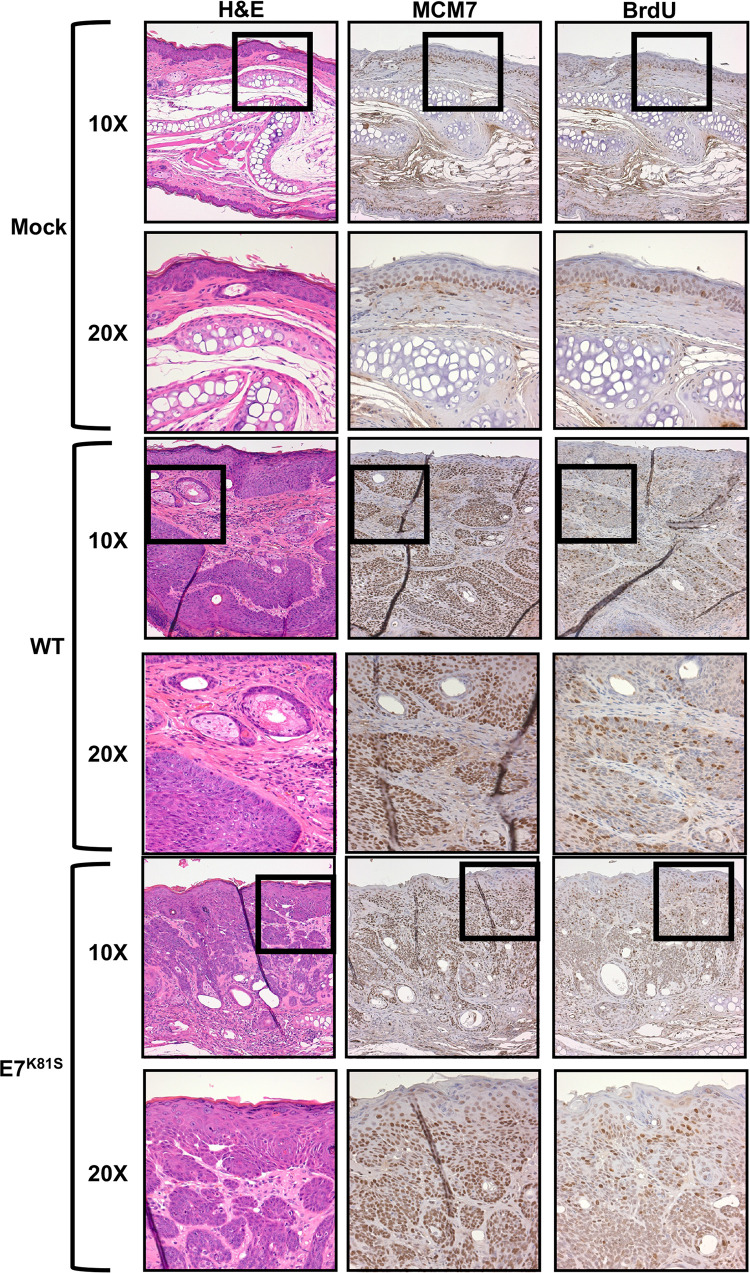
Similar expression of MCM7 and BrdU incorporation in the E7^K81S^- and WT induced lesions. FFPE tissues were subjected to immunohistochemistry (IHC) using antibodies against MCM7 and BrdU (as indicated). H&E and stained tissue sections were imaged at both a low (10X) and high (20X) magnification as indicated.

Two hallmarks of papillomavirus-induced lesions, including those caused by MmuPV1, are the induction of enhanced E2F transcription factor activity and the presence of cells within the suprabasal compartment of lesions that are supporting cellular DNA synthesis [45,52,66]. Previous studies with HPV16 and HPV18 E7 demonstrated that HPV E7’s ability to interact with or degrade PTPN14 does not alter E7’s ability to bind and inhibit pRB and is an pRB-independent function of high-risk HPV E7 [60,61]. Because the E7^K81S^ mutant-induced lesions arose later and were smaller at endpoint, we wanted to determine whether the E7^K81S^ mutant MmuPV1 was altered in its ability to increase cellular DNA synthesis or activate E2F. To do so, we assessed the incorporation of bromodeoxyuridine (BrdU) into newly synthesized DNA and expression of MCM7, an E2F-responsive gene, respectively. FFPE tissue slides were subjected to immunohistochemistry using antibodies against MCM7 and BrdU as previously described [66]. The E7^K81S^ mutant-induced lesions showed evidence of increased expression of MCM7 and suprabasal DNA synthesis, similar to lesions caused by wild type MmuPV1 infection (Fig 8). These results demonstrate that MmuPV1 E7’s interaction with PTPN14 is not required for MmuPV1 to cause unscheduled cellular DNA synthesis in suprabasal cells or to activate E2F.

### E7^K81S^ MmuPV1 is impaired in its ability to inhibit keratinocyte differentiation

**Fig 9:**
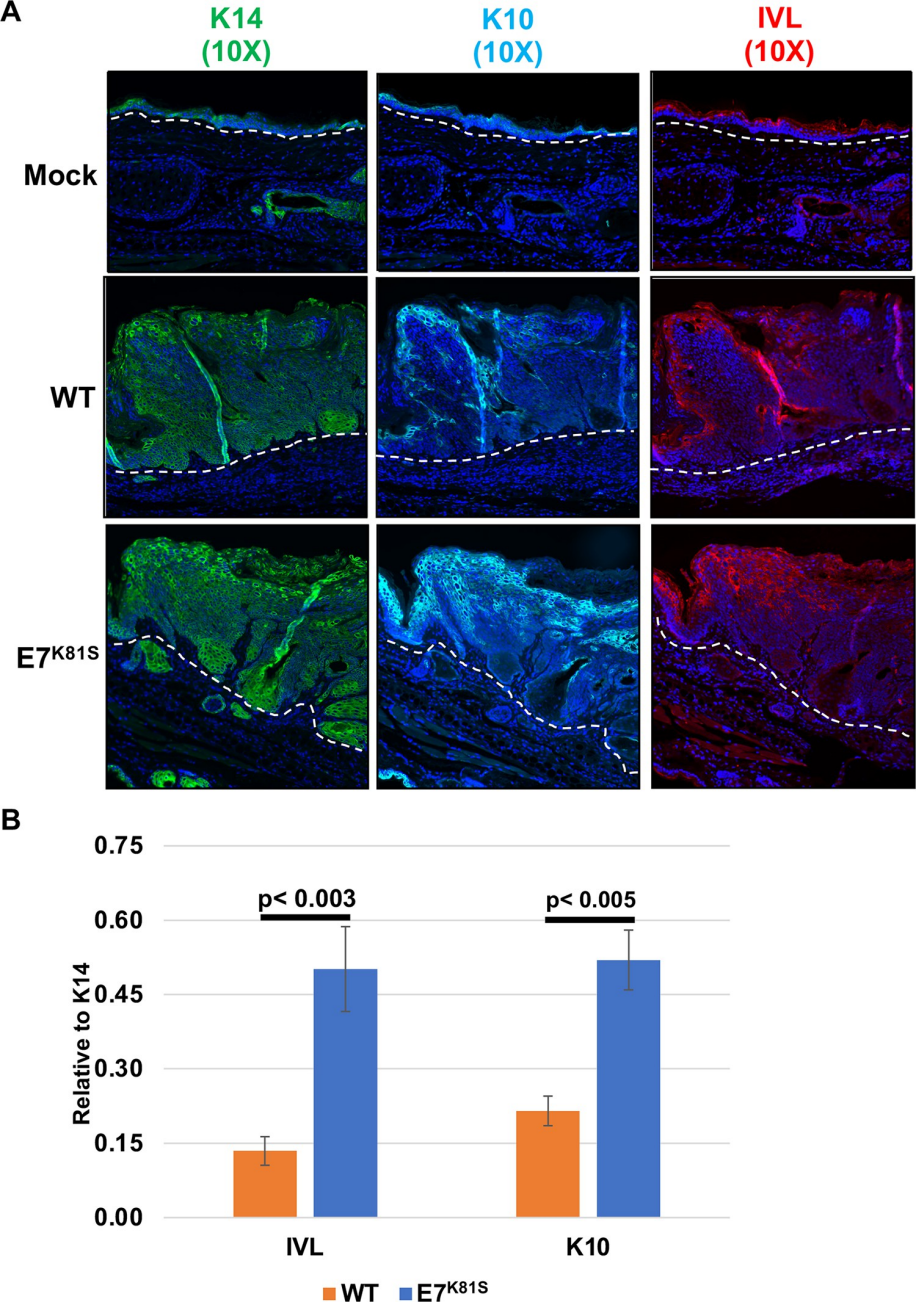
Keratinocyte differentiation is less impaired in the E7^K81S^-induced lesions than in WT MmuPV1-induced lesions. **(A)** FFPE tissue sections were subjected to Immunofluorescence (IF) using antibodies against K14 (green), K10 (cyan), and Involucrin (IVL, red). All sections were stained with the same secondary antibody (anti-mouse conjugated to AF488) and all images are pseudo colored for detection of AF-488. All images are 10X magnification. **(B)** IF images were quantified using ImageJ determining area with lesion that stained positive for K10 and IVL using 6 images per condition. K10 and IVL signal was normalized to K14 signal. Standard error is shown. Wilcoxon rank sum test was performed to assess statistical significance, and p-values are shown.

Keratinocyte differentiation is delayed in papillomavirus-induced lesions, including those caused by MmuPV1 [67]. E7 contributes to this delay in keratinocyte differentiation [60,68]. Therefore, we tested whether the interaction between MmuPV1 E7 and PTPN14 contributes to the virus’ ability to delay keratinocyte differentiation in *vivo*. To test this, we performed an immunofluorescence analysis on FFPE tissue slides using antibodies against Keratin 14 (K14) (a marker for basal keratinocytes), as well as Keratin 10 (K10) and Involucrin (IVL), which are markers for more differentiated keratinocytes. We observed similar hyperplasia of K14-positive keratinocytes in both the wild type and E7^K81S^ MmuPV1-induced lesions (Fig 9A), similar to what was seen before with wild type MmuPV1-induced lesions [38]. In wild type MmuPV1-induced lesions, K10 and IVL expression was restricted to the most superficial layers of the stratified squamous epithelium (Figs 9A and S7). In contrast, there was a significant expansion in expression of K10 (p < 0.005) and IVL (p < 0.003) in E7^K81S^ MmuPV1-induced lesions (Figs 9 and S7). These data indicate that MmuPV1 E7’s interaction with PTPN14 likely contributes to the ability of this papillomavirus to delay keratinocyte differentiation, a hallmark of papillomavirus-induced disease.

## Discussion

In this study, we demonstrate that MmuPV1 E7’s ability to interact with PTPN14 contributes to viral pathogenesis in mice and correlated with the ability of MmuPV1 to inhibit epithelial differentiation. MmuPV1 E7’s interaction with PTPN14 was initially identified in our previous AP/MS analyses with human cells [52]. *In silico* analysis of the amino acid sequence of MmuPV1 E7 compared to HPV18 E7 and other PV species suggested that MmuPV1 E7 interacts with PTPN14 using similar amino acids (Fig 1). We verified this interaction by IP/Western analysis in mouse cells and determined that amino acid K81 is critical for binding (Fig 2).

Using a virus carrying a mutation at amino acid K81 in MmuPV1 E7 (E7^K81S^) which impairs E7’s ability to bind PTPN14, we found that this mutant virus was able to cause lesions in nude mice at a similar frequency as wild type MmuPV1 E7 (Table 1). This result was not unexpected as the E7^K81S^ mutant virus was not impaired in its ability to infect or maintain its genome in mouse keratinocytes in 2D monolayer cell culture findings that suggest that this mutant virus is not deficient in early stage of the viral life (Fig 3). In infected animals, we determined that the E7^K81S^ mutant virus was proficient in viral gene replication, viral genome amplification, late protein production (L1 IF), and horizontal transmission (surrogate marker for infectious virus production) which are markers of the late stage of the viral life cycle (Figs 6 and 7). We also found that, consistent with the literature for HPV E7, MmuPV1’s ability to promote E2F activity and DNA replication (BrdU incorporation) was not dependent on MmuPV1 E7’s ability to interact with PTPN14 (Fig 8) [55,56,60].

However, E7^K81S^ mutant papilloma growth was reduced *in vivo*, with mutant papillomas arising later and reaching smaller sizes that were significantly different from WT papillomas at 6-months post-infection (Fig 4). Although the E7^K81S^ mutant-induced lesions developed severe dysplasia, none of the lesions in our studies showed early signs of the invasive disease that was present in WT-infected lesions (Fig 5). These impacts on viral pathogenesis correlated with an impairment in the ability of the E7^K81S^ MmuPV1 to delay keratinocyte differentiation (Fig 9). These findings lead us to conclude that E7’s ability to bind to the tumor suppressor PTPN14 contributes to pathogenesis. Our studies do not rule out, however, that the MmuPV1 E7^K81S^ mutant protein may also be defective for binding other, presently unknown cellular proteins and that these interactions may also contribute to the phenotypes that we observed in our studies with the MmuPV1 E7^K81S^ mutant. Therefore, it will be interesting to determine whether there are other cellular proteins that interact with MmuPV1 E7 through K81.

HPV E7 oncoproteins cause PTPN14 degradation by binding the ubiquitin ligase UBR4 (p600) [55,56]. MmuPV1 E7 also binds to UBR4 [52]. So far, we have not measured PTPN14 half-life and have only examined PTPN14 steady-state levels in transiently transfected cells (where not all cells are producing MmuPV1 E7). It will be important to conduct future experiments to determine whether and how MmuPV1 E7 reduces PTPN14 levels, perhaps by recruiting UBR4 to target PTPN14 for proteasome-mediated degradation. Future experiments could also test whether MmuPV1 E7’s interaction with UBR4 is important in viral pathogenesis and whether MmuPV1 encoding an E7 protein deficient in binding UBR4 has the same phenotype as the E7^K81S^ MmuPV1. A UBR4 binding-deficient mutant with a more severe phenotype than MmuPV1 E7^K81S^ might indicate that MmuPV1 E7 induces degradation of additional binding partners critical in viral pathogenesis.

PTPN14 is defined as a tumor suppressor because inactivating mutations in PTPN14 are associated with increased risk of some cancers [57,58,69]. A likely tumor suppressive activity of PTPN14 is its inhibition of YAP function [59,62]. PTPN14 has also been shown to be a p53 responsive gene that impairs cancer cell growth in pancreatic cancer in mice [69]. Since MmuPV1 E6 or E7 do not directly inhibit p53, it is possible that MmuPV1 E7’s interaction with PTPN14 may interfere with a function of p53 [34,38,52,70]. Additionally, PTPN14 has been implicated in regulating other pathways including inflammation, EGFR signaling, epithelial-to-mesenchymal transition, and survival of stem cells [58,71–78]. Further studies are needed to identify if MmuPV1 E7 alters any of these activities to promote viral pathogenesis.

In our studies, MmuPV1 E7’s ability to bind PTPN14 contributes to MmuPV1’s ability to inhibit epithelial cell differentiation *in vivo*. These findings are consistent with the observation that high-risk HPV E7 proteins inhibit the initial stages of differentiation in 2D cell culture models, dependent on their ability to degrade PTPN14 [60]. In 3D raft culture models HPV E7-mediated PTPN14 degradation contributes to maintenance of the basal cell identity of HPV positive cells but does not suppress overall tissue stratification [62]. Similarly, a recent study with 2D cell culture models provided evidence that MmuPV1 E6, not MmuPV1 E7, promotes basal cell identity [79]. Hence it will be interesting to determine whether there might be differences in the activities of PTPN14 that MmuPV1 E7 targets compared to the high-risk HPV E7 oncoproteins [60,62]. Cutaneous HPV E7 bind UBR4 and PTPN14, although both UBR4 and PTPN14 are bound less efficiently by cutaneous HPV E7 than by mucosal HPV E7, and cutaneous HPV E7 have minimal effects on the steady-state level of PTPN14 [55,63,80]. It will be interesting to determine if inhibition of PTPN14 by MmuPV1 and cutaneous HPV E7 proteins may have different biological consequences than PTPN14 inhibition by high-risk mucosal HPV E7 proteins. Alternatively, the consequences of PTPN14 inhibition may be different in cutaneous and mucosal epithelia. Given that MmuPV1 can also infect mucosal tissues it will be interesting to perform cervicovaginal infections with the WT and E7^K81S^ virus to determine the importance of this interaction during a MmuPV1 mucosal infection.

We have previously shown that the cutaneous β-HPV8 and MmuPV1 E6 proteins inhibit keratinocyte differentiation through its interactions with MAML1 as well as SMAD2 and SMAD3, which are critical co-activators of NOTCH and TGF-β signaling, respectively [38]. Therefore, MmuPV1 encodes multiple mechanisms by which it impairs the differentiation process. It will be interesting to determine whether this is also observed with cutaneous HPVs. Further studies are clearly warranted to investigate the contributions of MmuPV1 and cutaneous HPV E6 and E7 proteins to the inhibition of differentiation and to maintain basal cell identity as well as the relative importance of these activities in viral pathogenesis.

## Materials and methods

### Cells

NIH 3T3 murine fibroblasts were obtained from ATCC and grown in DMEM supplemented with 10% calf serum. Mouse keratinocytes were isolated from the skin of neonate pups from the FVB/N and C57/Black6 backgrounds. After incubation in phosphate-buffered saline (PBS) containing 10% antibiotics for 2 min, skin pieces were incubated in 0.25% trypsin overnight at 4°C. The epidermis was then separated from the dermis using sterile forceps, minced with a single-edge razor blade, and then stirred for 1 h at 37°C in F-medium to generate a single-cell suspension. The cells were strained using 0.7-mm membrane (102095–534; VWR) and cultured in F-medium containing 10 mM Y-27632 Rho-kinase inhibitor [81] in the presence of mitomycin C (M4287; Sigma)-treated 3T3 J2 fibroblasts. Early passage cells were transfected in Keratinocyte Serum-free media (1074–001, Gibco) with dsRed alone or in combination with WT or mutant MmuPV1 genomes using the Lipofectamine 3000 system (L3000-008, Invitrogen). Transfected cells were treated with G418 48 hours post-transfection. Following selection, keratinocytes were cultured in F-medium containing Y-27632.

### Plasmids

N-terminally and C-terminally Flag/HA tagged MmuPV1 E7 and HPV16 E7 were cloned into pCMV Bam/neo vectors [82]. The MmuPV1 E7^K81S^ mutant was PCR amplified with primers containing the K81S mutation and Q5 High Fidelity Polymerase (NEB). The PCR product was treated with DpnI (NEB) for 1 hour, then transformed into 10-Beta competent E. coli cells (NEB).

### IP and Immunoblot analysis

NIH3T3 cells were transfected using Lipofectamine 2000 (Invitrogen, ThermoFisher). At 48 hr post-transfection cells were harvested in EBC buffer (50mM Tris-Cl pH 8.0, 150mM NaCl, 0.5% NP-40 and 0.5mM EDTA) supplemented with protease inhibitors (Pierce). Anti-Hemagglutinin (HA; Sigma) antibodies coupled to agarose beads were used for immunoprecipitations followed by SDS-PAGE and western blot analysis on PVDF membranes. After incubation with appropriate primary and secondary antibodies, blots were visualized by enhanced chemiluminescence, and images captured using a Syngene ChemiXX6 imager with Genesys software version 1.5.5.0. Signals were quantified with Genetools software version 4.03.05.0. Antibodies included PTPN14 (CST #13808), HA (Abcam ab9110), Actin (Millipore MAB1501), pRB (Santa Cruz SC74570), Flag (Sigma F3165), and GFP (Santa Cruz SC-9996). Secondary anti-mouse and anti-rabbit HRP antibodies were from GE Healthcare.

### Alignment of E7 oncogenes

E7 protein sequences were retrieved from PaVE (https://pave.niaid.nih.gov), aligned by MAFFT FFT-NS-2 (v7.453), and visualized in Jalview. Sequences of PTPN14 orthologs were retrieved from the NCBI Gene database (https://www.ncbi.nlm.nih.gov/gene), in which orthologs were calculated using NCBI’s Eukaryotic Genome Annotation pipeline. Sequences were aligned with COBALT and visualized in Jalview. Three-dimensional alignment was performed in PyMOL using the deposited structure 6IWD [63]. I-TASSER was used for prediction of the MmuPV1 E7 structure [83–85].

### Animals

Immunodeficient athymic nude-FoxN1nu/nu mice were purchased from Envigo. Mice were housed in the Association for Assessment of Laboratory Animal Care-approved Clinical Sciences Center Vivarium. All procedures were carried out in accordance with an animal protocol approved by the University of Wisconsin Institutional Animal Care and Use Committee (IACUC; protocol number M005871).

### Infection of nude mice with MmuPV1 quasivirus

MmuPV1 quasivirus (WT, E7^D90A^, and E7^K81S^; the term “quasivirus” is used in the papillomavirus field to describe virus generated *in vitro* after transfecting in recircularized viral genome with a plasmid that expresses the L1 and L2 proteins in 293 cells) was generated as described before [38,52,64]. Briefly, 293 FT cells were co-transfected using Lipofectamine 2000 (11668–019, Invitrogen) with recircularized viral genome, either WT or mutant, and the MmuPV1 pShell vector (encodes the L1 and L2 capsid proteins). After incubation at 37oC for 48 hr, cells were harvested, and viral extracts generated [64]. Virus titers were quantified via Southern blot analysis which allowed us to determine viral genome equivalent (VGE) to determine viral concentration in each virus prep. Additionally, we tested infectivity of each virus prep by infecting JB6 cells with equal volume of each virus prep. RNA was isolated 72 hr post-infection and used to perform RT-PCR for the E1^E4 viral transcript (E1ˆE4-forward, 5’-CATTCGAGTCACTGCTTCTGC-3’; E1ˆE4-reverse, 5’-GATGCAGGTTTGTCGTTCTCC-3’). Infectious quasivirus was used to infect Nude-FoxN1^nu/nu^ mice using 10^8^ VGE of each virus prep for infected animals and PBS for mocks as previously described [38,52]. Briefly, mice were anaesthetized and ears were scarified using a 27-gauge needle. Scarified ears were treated with a volume equivalent to 10^8^ VGE with both ears being infected for a total of 2 sites per animal. Lesion development was monitored biweekly for 4–6 months post-infection.

### Southern blot analysis for viral genome

Total genomic DNA was extracted from transfected mouse keratinocytes using Qiagen’s DNeasy Blood and Tissue kit. A total of 2 μg DNA was digested with BamHI, an enzyme that will linearize the MmuPV1 genome, and DpnI, an enzyme that will digest unreplicated, bacterial input DNA. These digested DNAs were electrophoresed on a 0.8% agarose gel along with an MmuPV-1-containing plasmid that was digested with BamHI to release the viral genome as a standard. After electrophoresis, these DNAs were transferred to the Hybond N+ membrane (GE Healthcare, Amersham, Buckinghamshire). The membrane was then probed with a set of 20 oligos complementary and specific to MmuPV1 that were labeled with γ-32P-ATP. To visualize MmuPV-1 DNA, the washed membrane was exposed to a PhosphorImager screen that was then scanned using a Typhoon (GE Healthcare). For titering virus prep, 10 μL of virus prep was treated with equal volume virus release buffer (1 mg/mL Proteinase K, 0.5% SDS, and 25mM EDTA in water) and incubated at 56°C for 1 hr. Samples were subjected to similar analysis as described above starting at gel electrophoresis.

### qPCR for viral genome copy number

FVB cells transfected with recircularized viral genome were harvested following selection and outgrowth and DNA was isolated using Qiagen Blood and Tissue kit (69506) following manufacturer’s instructions. Isolated DNA was then used for qPCR analysis using E2 primers (MmuPV1_E2_1 5’-GCCCGAAGACAACACCGCCACG-3’ and MmuPV1_E2_2 5’-CCTCCGCCTCGTCCCCAAATGG-3’) to detect viral genomes and murine 18S primers (18S_1 5’-CGCCGCTAGAGGTGAAATTC-3’ and 18S_2 5’-TTGGCAAATGCTTTCGCTC-3’) to approximate genomic DNA concentration in each reaction [44]. qPCR was performed using SYBR green system from Qiagen (204143) following manufacturer’s protocol and used the Bio-Rad CFX96 machine for detection. To determine viral genome, copy number and cellular DNA concentration, a ladder was made for both genomes copy number (10^8^ to 10^2^ using 100-fold dilutions) and cellular DNA concentration (10 μg to .01 μg using 10-fold dilutions) to generate Ct values for viral genome copy number and concentration of cellular DNA. Ct values for specific samples were converted to viral copy number or cellular DNA concentration present within each sample. Viral copy number was normalized to cellular DNA concentration and relative copy number was determined comparing each sample to the average copy number/cellular DNA for WT transfected cells.

### BrdU incorporation and tissue collection

One hour prior to euthanasia, infected mice were injected intraperitoneally with 250 μL of 12.5 mg/mL bromodeoxyuridine (BrdU, Sigma). Tissues were fixed in 4% paraformaldehyde for 24 hr and then switched to 70% EtOH. Fixed tissues were processed, embedded in paraffin, and 5-μm sections generated. Every 10^th^ section was stained with Hematoxylin and Eosin (H&E) for histological analysis.

### Immunofluorescence and Immunohistochemistry

Immunofluorescence was performed on Formalin-fixed paraffin-embedded tissue sections for Keratin 14 (K14), Keratin 10 (K10), and Involucrin (Ivl). Briefly, sections were deparaffinized in Xylene and rehydrated in 100%, 95%, 70%, and 50% EtOH and then in PBS. Rehydrated sections were then treated with boiling Tris-EDTA pH10 antigen retrieval buffer for 20 mins. Sections were blocked with 5% Milk containing goat serum for 1 hr at RT and then primary antibody added to incubate overnight. Next day, sections were treated with secondary antibody following washes for 1hr using goat-α-rabbit AF488. Sections were then washed and subject to Hoechst stain for 10 mins before mounting. K14 and L1 capsid protein co-staining was performed as previously described using the tyramide-based signal amplification (TSA) method [86]. A detailed protocol can be found at https://www.protocols.io/view/untitled-protocol-i8cchsw. For immunohistochemistry, slides were deparaffinized and rehydrated as described above. Antigen retrieval was performed on rehydrated sections using citrate buffer (pH 6) for 20 min by heating in a microwave (3 minutes on max and 17 minutes at 70% power). Blocking was performed using 2.5% horse serum in PBS for 1 hr at room temperature (RT). Slides were then treated with primary antibody overnight in a humidified chamber. Vectastain ABC (HRP) kit (PK4000, Vector) was used to detect signal. Following primary antibody treatment, slides were washed and then treated with universal secondary antibody for 1 hr at RT. Following secondary antibody treatment, slides were treated with an equal mix of A and B reagents for 1 hr at RT. Slides were then treated with DAB for signal development and subsequently counterstained with Hematoxylin (H-3404, Vector). Slides were dehydrated using the EtOH concentrations described above but in reverse order, followed by Xylene washes before being mounted. All images were taken using a Zeiss AxioImager M2 microscope using AxioVision software, version 4.8.2. Primary antibodies used include L1 (gift from Chris Buck), K14 (905301, BioLegend), K10 (PRB-159P, Covance), Involucrin (PRB-140C, Covance), MCM7 (clone DCS-141, MA5-14291, Invitrogen), and BRDU (Clone Ab3, NA61, Millipore Sigma). Secondary antibodies include anti-Rb-AF488 (A11-034, Invitrogen) and anti-SA-AF647 (S32357, Invitrogen). IF images were quantified using ImageJ where areas of positive staining were determined by total area times the percent of the area that had signal. Values for L1, K10, and IVL were normalized to K14 in the analysis. Workflow is provided in S8 Fig

### RNA/DNA In situ hybridization

To detect MmuPV1 E4 transcripts in the cytoplasm and MmuPV1 DNA genomes in the nucleus, we used RNAscope 2.5 HD Assay-Brown (Advanced Cell Diagnostics, Newark, CA) and a MmuPV1 E4-specific probe set (Catalog #473281) according to manufacturer’s instructions as previously described [45,51]. To detect viral DNA alone, we pretreated slides with RNAse, as previously described [45,51]. Quantification of the fraction of infected cells going through viral genome amplification was determined using an ImageJ plugin developed by the lab of Dr. David Ornelles (Wake Forest University).

### Statistics

All statistical tests were done using MStat software (https://oncology.wisc.edu/mstat/).

## Supporting information

S1 Fig Alignment of primate and murine Papillomavirus E7’s and PTPN14. **(A)** Amino acid sequence alignment of the C-terminus of several primate, rodent, and bovine papillomavirus E7 proteins. R84, and L91 are two amino acids in HPV18 E7 that make contact with PTPN14 as determined by the HPV18 E7-PTPN14 crystal structure [63]. Identity with R84, and L91 is indicated in red. An arginine corresponding to HPV18 R84 is conserved among many papillomavirus E7 but is replaced by lysine in two rodent papillomavirus E7 (yellow highlight). **(B)** Amino acid sequence alignment of a segment of the PTP domain of selected primate, rodent, and bovine PTPN14 proteins. F1044, G1055, and E1095 are three amino acids in human PTPN14 that make contact with HPV18 E7 as determined by the HPV18 E7-PTPN14 crystal structure [63], and these are highlighted in red. **(C)** Additional structure image of MmuPV1 E7 (yellow) and PTPN14 (silver) and how they may interact. Lysine 81 is highlighted in red. **(D)** Ribbon structures of HPV18 E7 (blue) and MmuPV1 E7 (yellow) overlayed to determine similarity in secondary structure. Lysine 81 in MmuPV1 E7 is highlighted in red.
(TIFF)

S2 Fig Immunoprecipitation analysis of PTPN14 steady state levels from multiple independent experiments. Experiment 1 images correspond to the data shown in the left panel of Fig 2.
(TIF)

S3 Fig E7^K81S^ mutant MmuPV1 maintains genome and can generate infectious quasivirus. **(A)** Unspliced southern blot showing all conditions run on gel image shown in Fig 3. (**B**) Total RNA was isolated from JB6 mouse epithelial cells 48hrs post-infection with WT MmuPV1, E7^D90A^, and E7^K81S^ quasivirus preps. cDNA was generated and subjected to RT-PCR to determine if quasivirus prep was infectious using primers that target the E1^E4 viral transcript.
(TIF)

S4 Fig Sequence verification of MmuPV1 E7 in lesions infected with the E7^K81S^ mutant genome. DNA was isolated from FFPE tissue sections and then subjected to PCR for the MmuPV1 E7 gene. PCR products were sent for sequencing to verify presence of the E7^K81S^ mutation. A representative sequencing result is shown.
(TIF)

S5 Fig Mouse Ear Images comparing size of warts. Representative images of mouse ears for Mock, WT, and E7^K81S^ mutant are shown. Lesions are located within the circle.
(TIF)

S6 Fig L1 capsid staining in the fully differentiated squamous epithelia. FFPE tissue sections subjected to IF analysis for K14 (green) and L1 (orange) where L1 staining is solely detected in K14 negative differentiated cells. All images were taken at 10X magnification. Dashed white lines indicate the basement membrane of epithelial tissues.
(TIF)

S7 Fig Differentiation marker staining of cutaneous lesions in E7^K91S^ and WT infected animals. Representative images of FFPE tissue sections subjected to IF analysis using antibodies against K14 (green), K10 (cyan), and IVL (red) for WT- and E7^K81S^-infected animals. All images were taken at 10X magnification. Dashed white lines indicate basement membrane of epithelial tissues.
(TIF)

S8 Fig Schema for IF quantification. The workflow that was used for IF quantification of differentiation markers is shown.
(PDF)
