# Supplementary figures and images for: MmuPV1 E7’s interaction with PTPN14 delays Epithelial differentiation and contributes to virus-induced skin disease
Source: PLoS Pathog. 2023 Apr 10;19(4):e1011215. doi: 10.1371/journal.ppat.1011215 (PMC10085053; doi:10.1371/journal.ppat.1011215)

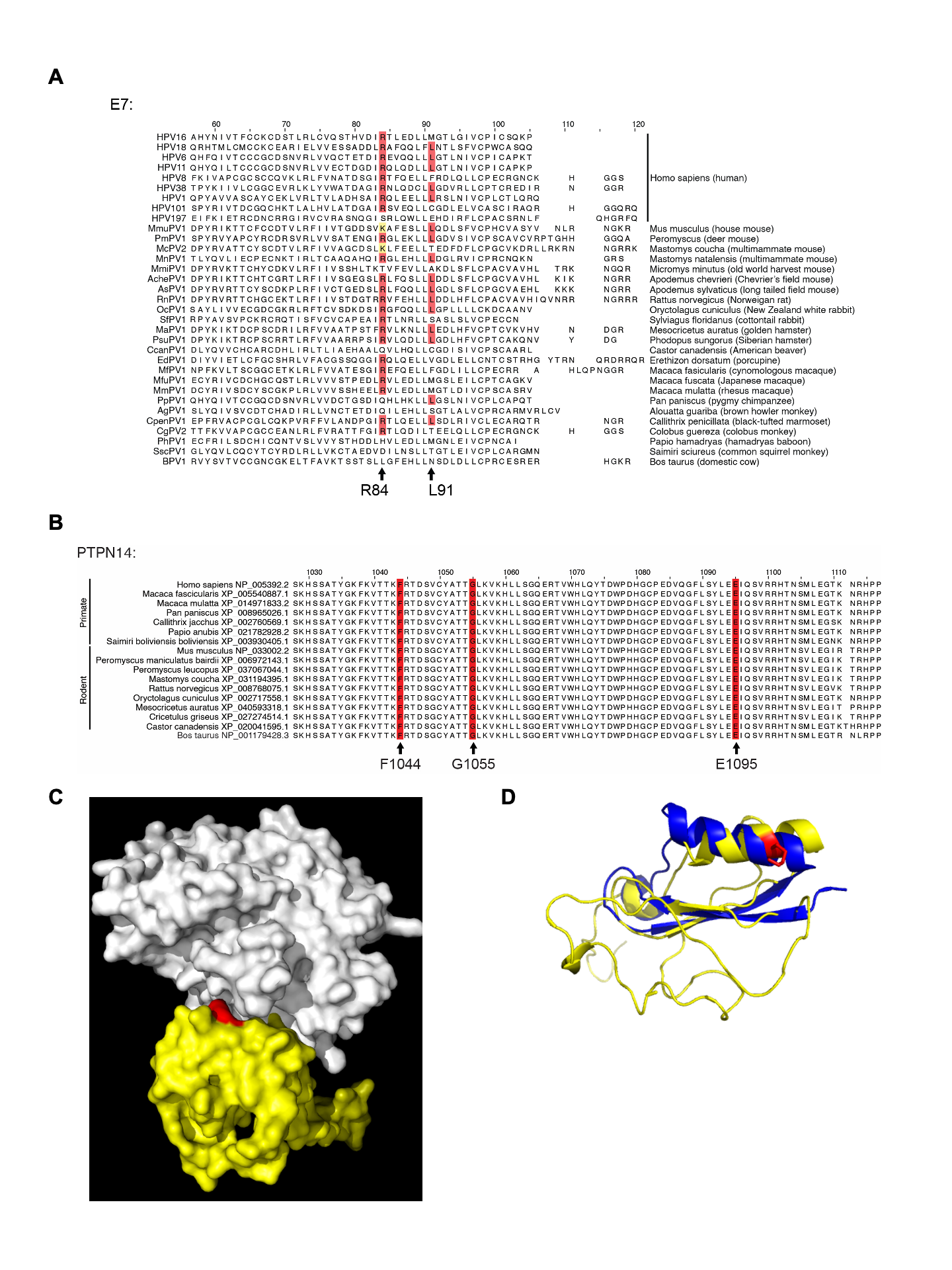

Supplement: S1 Fig — (A) Amino acid sequence alignment of the C-terminus of several primate, rodent, and bovine papillomavirus E7 proteins. R84, and L91 are two amino acids in HPV18 E7 that make contact with PTPN14 as determined by the HPV18 E7-PTPN14 crystal structure [63]. Identity with R84, and L91 is indicated in red. An arginine corresponding to HPV18 R84 is conserved among many papillomavirus E7 but is replaced by lysine in two rodent papillomavirus E7 (yellow highlight). (B) Amino acid sequence alignment of a segment of the PTP domain of selected primate, rodent, and bovine PTPN14 proteins. F1044, G1055, and E1095 are three amino acids in human PTPN14 that make contact with HPV18 E7 as determined by the HPV18 E7-PTPN14 crystal structure [63], and these are highlighted in red. (C) Additional structure image of MmuPV1 E7 (yellow) and PTPN14 (silver) and how they may interact. Lysine 81 is highlighted in red. (D) Ribbon structures of HPV18 E7 (blue) and MmuPV1 E7 (yellow) overlayed to determine similarity in secondary structure. Lysine 81 in MmuPV1 E7 is highlighted in red. (TIFF) [file ppat.1011215.s001.tiff]

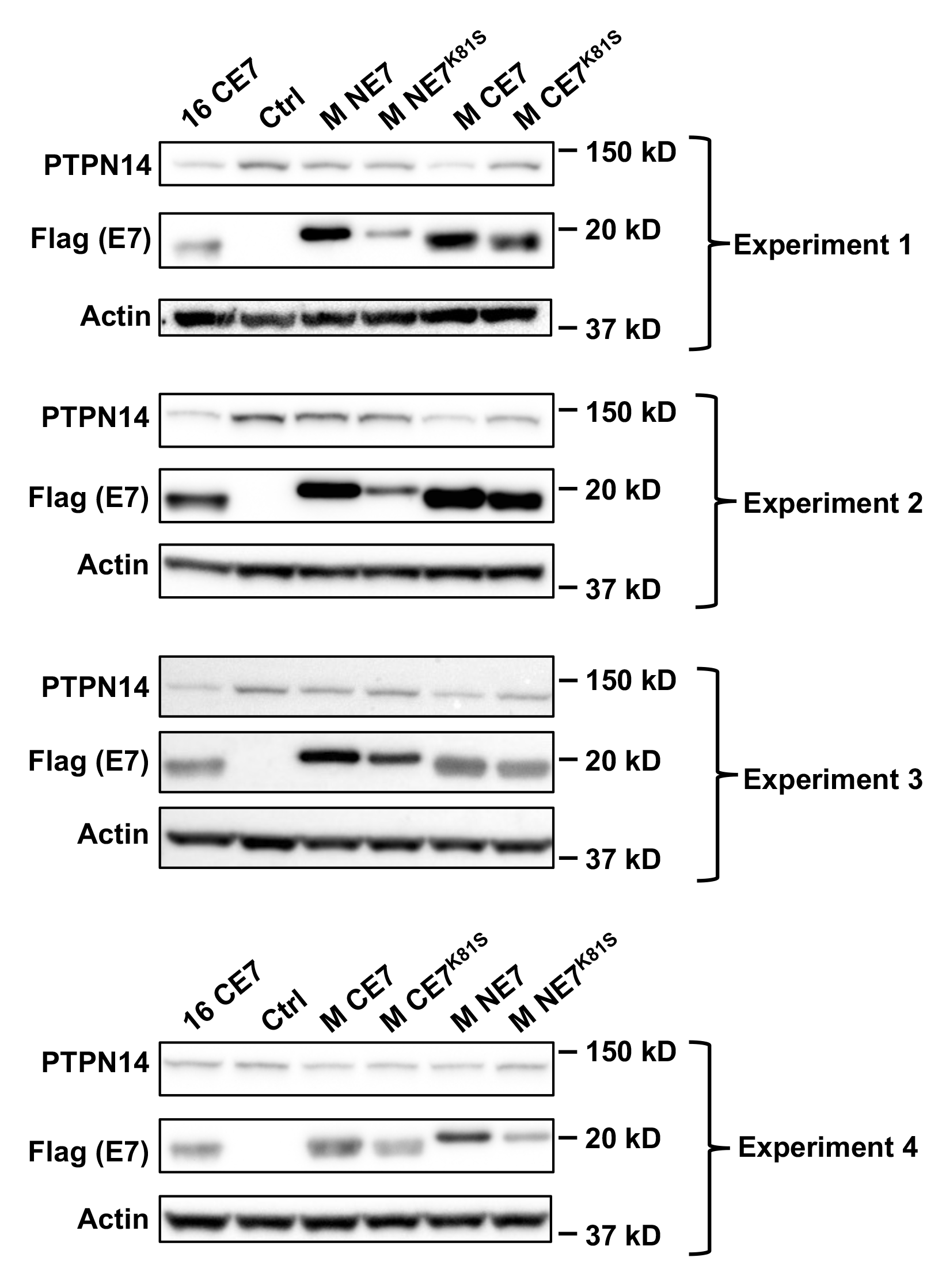

Supplement: S2 Fig — Experiment 1 images correspond to the data shown in the left panel of Fig 2. (TIF) [file ppat.1011215.s002.tif]

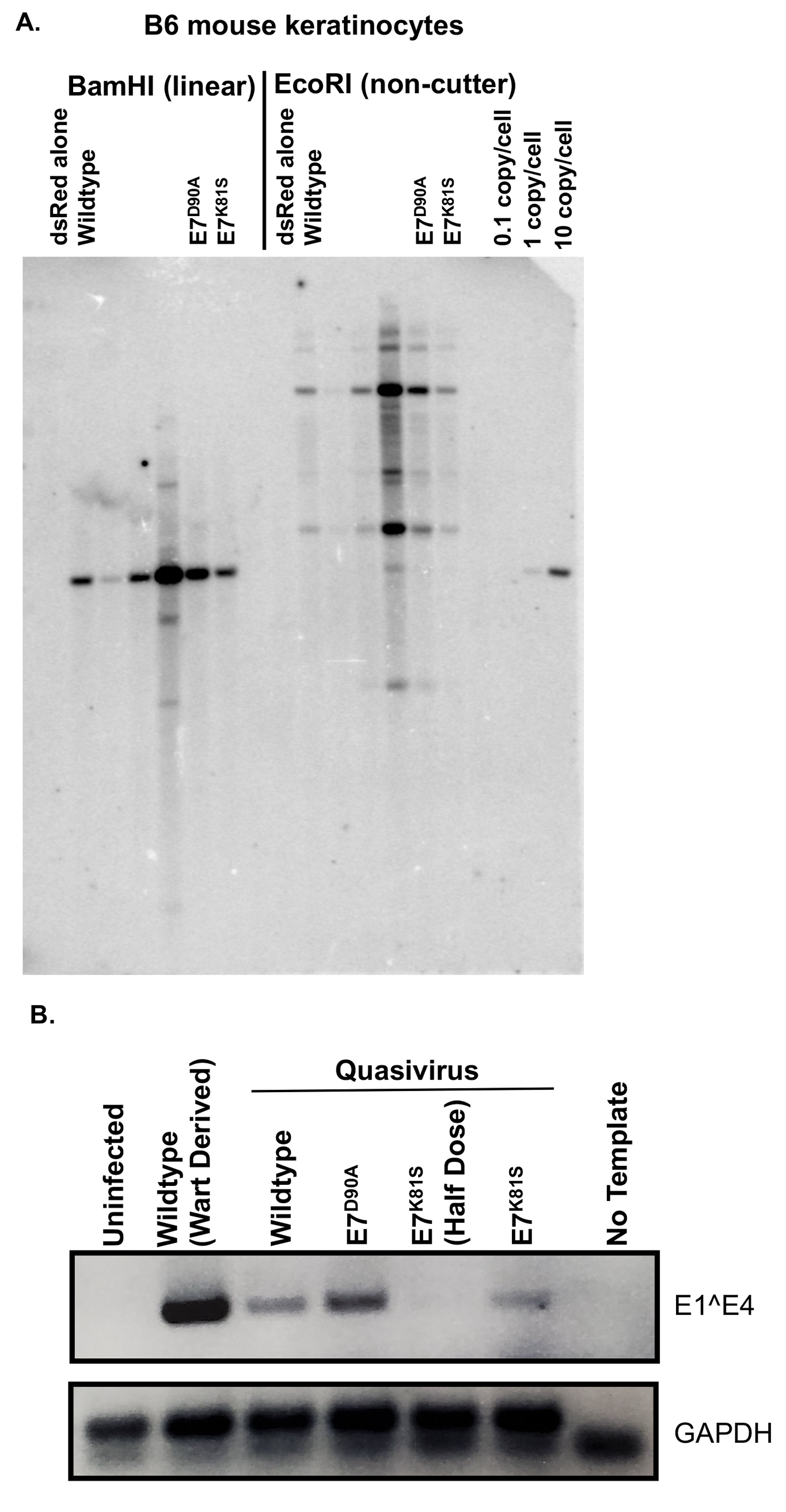

Supplement: S3 Fig — (A) Unspliced southern blot showing all conditions run on gel image shown in Fig 3. (B) Total RNA was isolated from JB6 mouse epithelial cells 48hrs post-infection with WT MmuPV1, E7D90A, and E7K81S quasivirus preps. cDNA was generated and subjected to RT-PCR to determine if quasivirus prep was infectious using primers that target the E1^E4 viral transcript. (TIF) [file ppat.1011215.s003.tif]

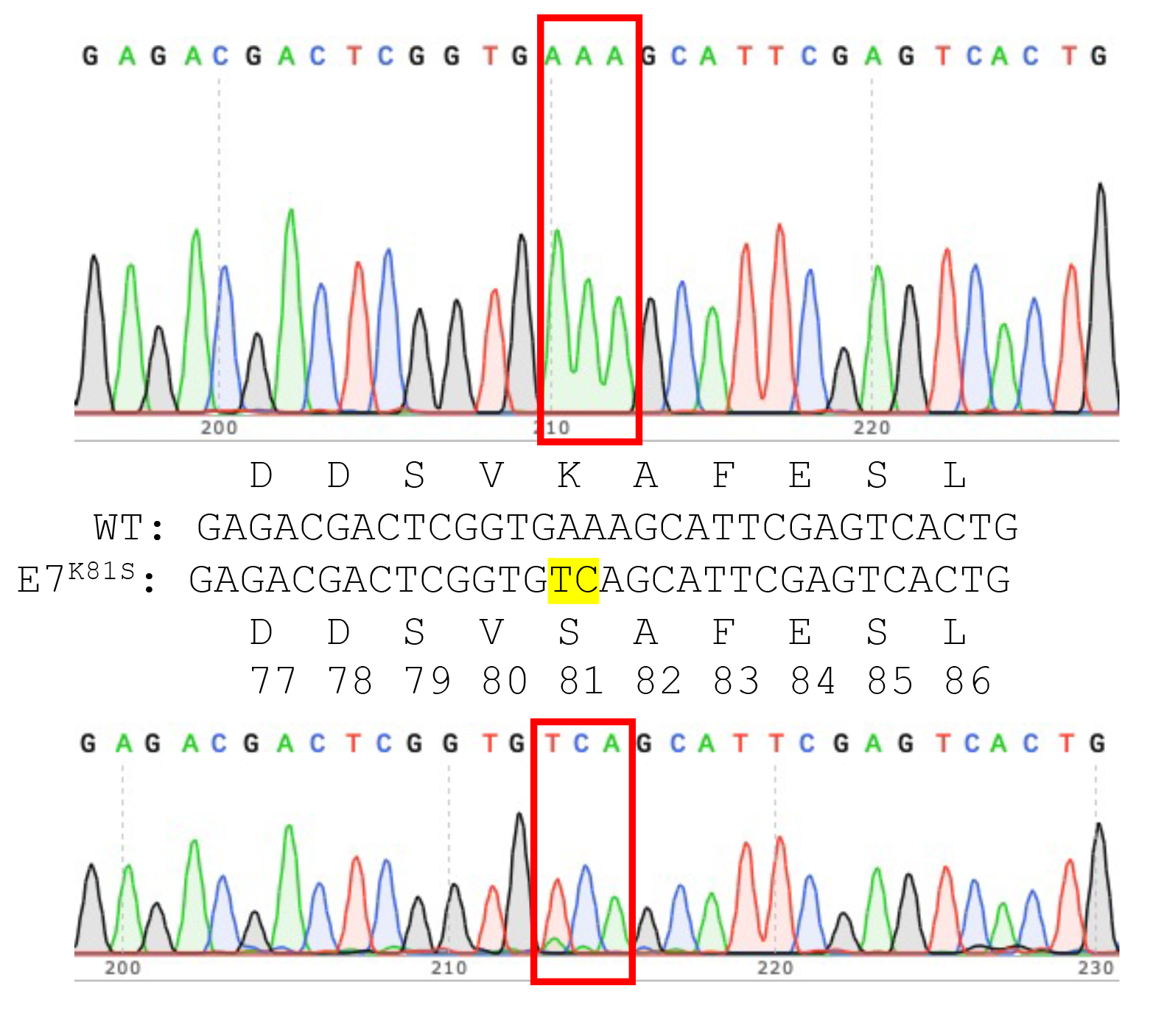

Supplement: S4 Fig — DNA was isolated from FFPE tissue sections and then subjected to PCR for the MmuPV1 E7 gene. PCR products were sent for sequencing to verify presence of the E7K81S mutation. A representative sequencing result is shown. (TIF) [file ppat.1011215.s004.tif]

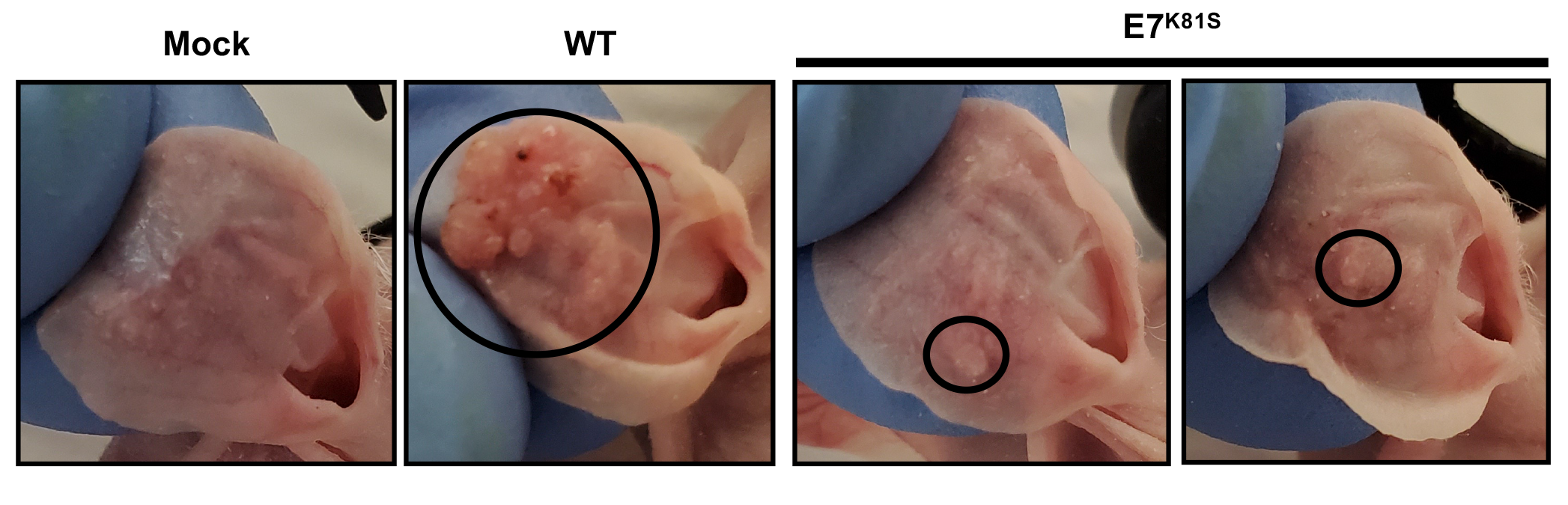

Supplement: S5 Fig — Representative images of mouse ears for Mock, WT, and E7K81S mutant are shown. Lesions are located within the circle. (TIF) [file ppat.1011215.s005.tif]

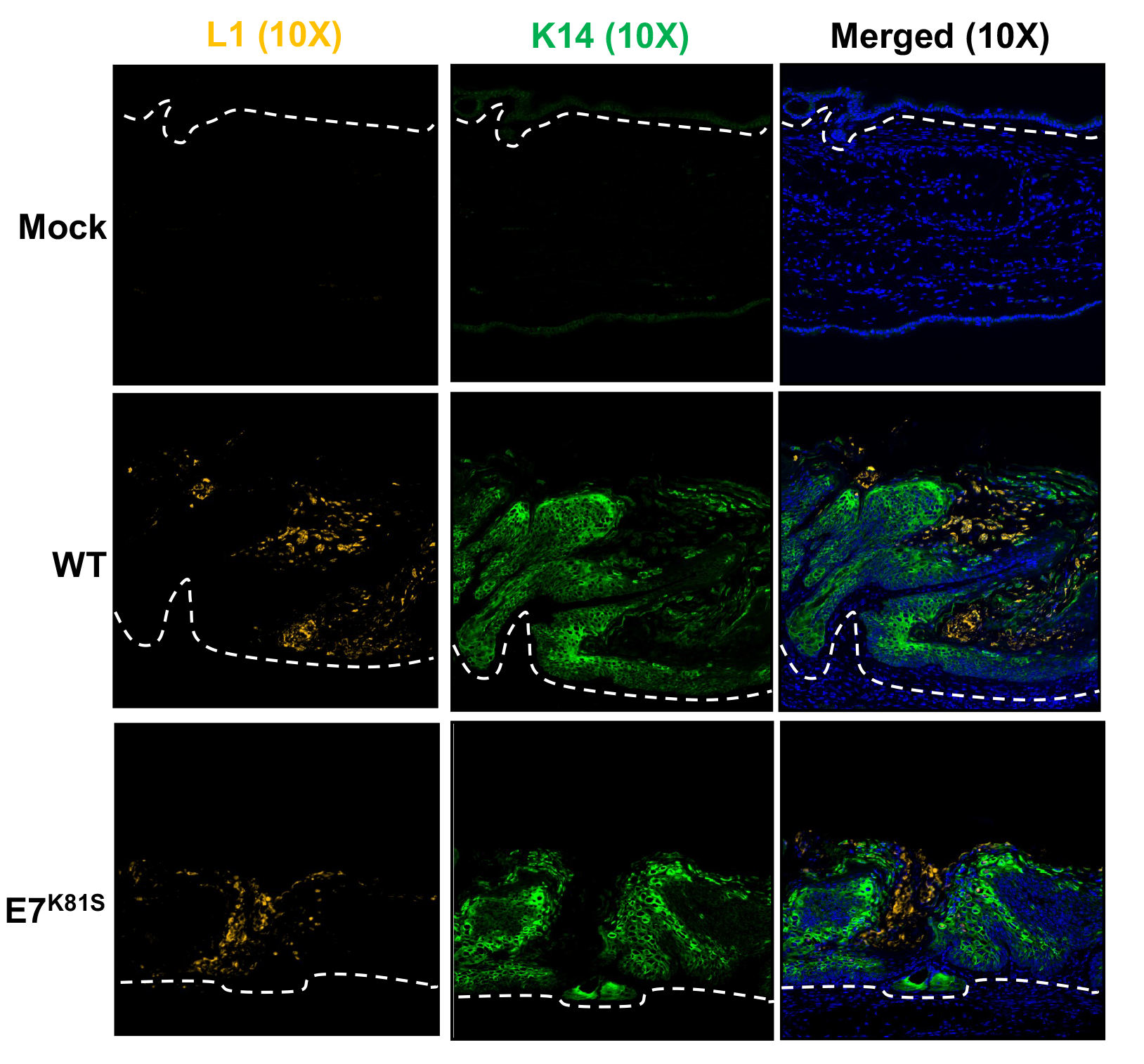

Supplement: S6 Fig — FFPE tissue sections subjected to IF analysis for K14 (green) and L1 (orange) where L1 staining is solely detected in K14 negative differentiated cells. All images were taken at 10X magnification. Dashed white lines indicate the basement membrane of epithelial tissues. (TIF) [file ppat.1011215.s006.tif]

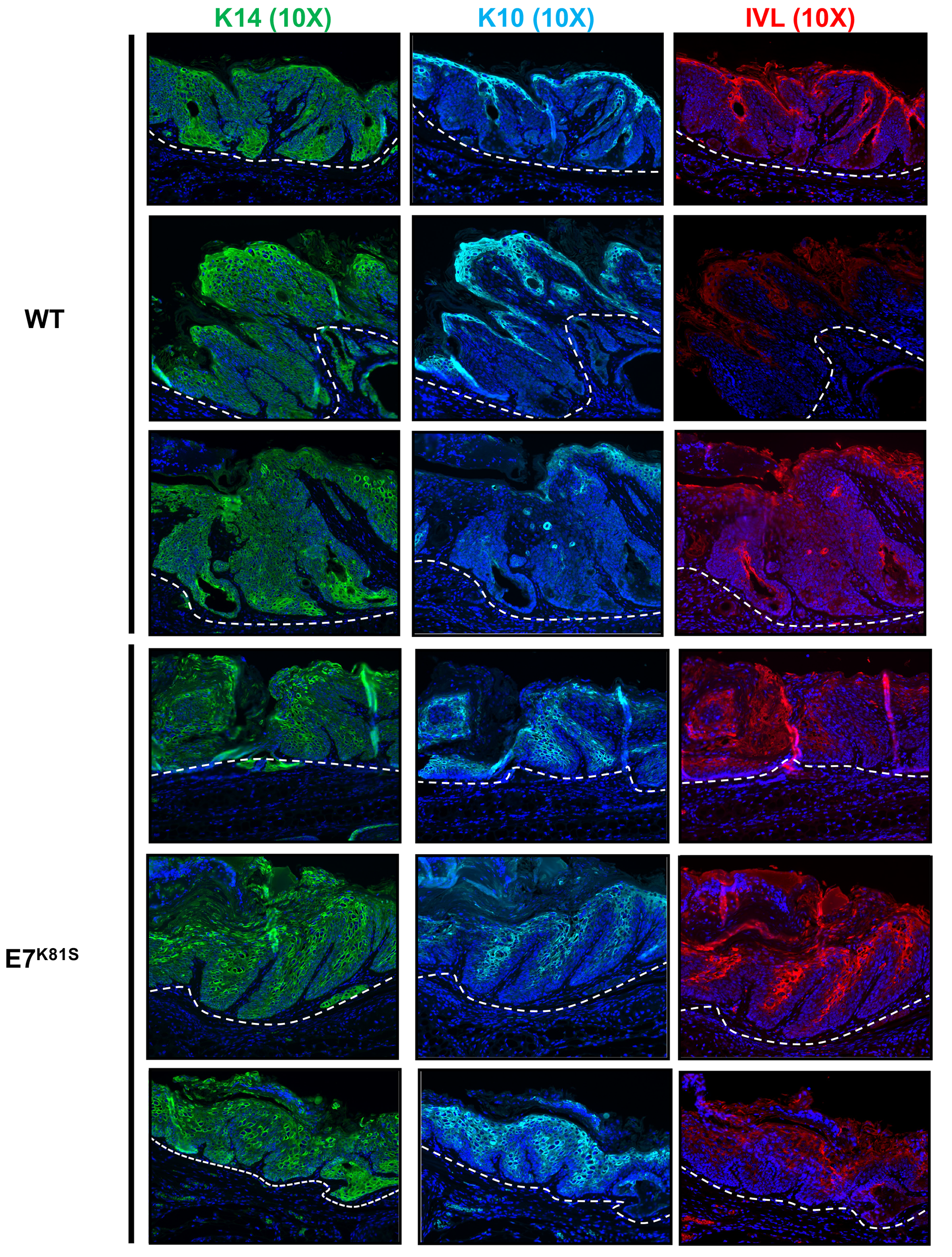

Supplement: S7 Fig — Representative images of FFPE tissue sections subjected to IF analysis using antibodies against K14 (green), K10 (cyan), and IVL (red) for WT- and E7K81S-infected animals. All images were taken at 10X magnification. Dashed white lines indicate basement membrane of epithelial tissues. (TIF) [file ppat.1011215.s007.tif]

Figure S8

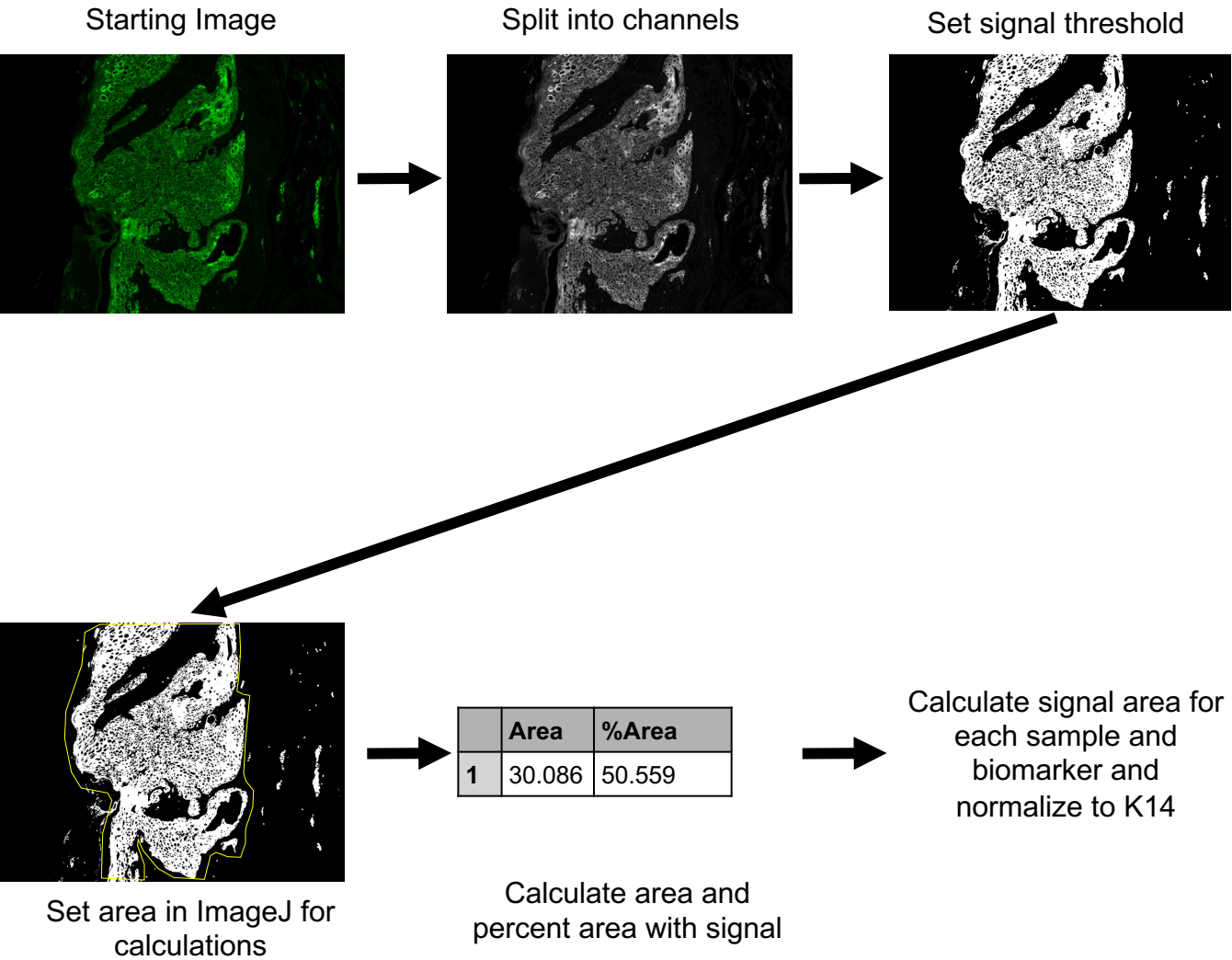

Supplement: S8 Fig — The workflow that was used for IF quantification of differentiation markers is shown. (PDF) [file ppat.1011215.s008.pdf]
